# Multi-omic approach characterises the neuroprotective role of retromer in regulating lysosomal health

**DOI:** 10.1038/s41467-023-38719-8

**Published:** 2023-05-29

**Authors:** James L. Daly, Chris M. Danson, Philip A. Lewis, Lu Zhao, Sara Riccardo, Lucio Di Filippo, Davide Cacchiarelli, Daehoon Lee, Stephen J. Cross, Kate J. Heesom, Wen-Cheng Xiong, Andrea Ballabio, James R. Edgar, Peter J. Cullen

**Affiliations:** 1 School of Biochemistry, Biomedical Sciences Building, University Walk, University of Bristol, Bristol, BS8 1TD UK; 2 Bristol Proteomics Facility, School of Biochemistry, Biomedical Sciences Building, University Walk, University of Bristol, BS8 1TD Bristol, UK; 3 Department of Neurosciences, Case Western Reserve University, Cleveland, OH USA; 4 Telethon Institute of Genetics and Medicine, Armenise/Harvard Laboratory of Integrative Genomics, Pozzuoli, Italy; 5 Next Generation Diagnostic srl, Pozzuoli, Italy; 6 Department of Translational Medicine, University of Naples “Federico II”, Naples, Italy; 7 School for Advanced Studies, University of Naples “Federico II”, Naples, Italy; 8 Wolfson Bioimaging Facility, Faculty of Biomedical Sciences, University of Bristol, Bristol, UK; 9 Department of Molecular and Human Genetics and Neurological Research Institute, Baylor College of Medicine, Houston, TX USA; 10 Department of Pathology, Cambridge University, Tennis Court Road, Cambridge, UK; 11 Present Address: Department of Infectious Diseases, School of Immunology and Microbial Sciences, Guy’s Hospital, King’s College London, SE1 9RT, London, UK

**Keywords:** Lysosomes, Protein-protein interaction networks, Neurodegeneration

## Abstract

Retromer controls cellular homeostasis through regulating integral membrane protein sorting and transport and by controlling maturation of the endo-lysosomal network. Retromer dysfunction, which is linked to neurodegenerative disorders including Parkinson’s and Alzheimer’s diseases, manifests in complex cellular phenotypes, though the precise nature of this dysfunction, and its relation to neurodegeneration, remain unclear. Here, we perform an integrated multi-omics approach to provide precise insight into the impact of Retromer dysfunction on endo-lysosomal health and homeostasis within a human neuroglioma cell model. We quantify widespread changes to the lysosomal proteome, indicative of broad lysosomal dysfunction and inefficient autophagic lysosome reformation, coupled with a reconfigured cell surface proteome and secretome reflective of increased lysosomal exocytosis. Through this global proteomic approach and parallel transcriptomic analysis, we provide a holistic view of Retromer function in regulating lysosomal homeostasis and emphasise its role in neuroprotection.

## Abstract (web summary)

Daly, Danson and colleagues employ a multi-omic approach in neuroglioma cells to characterise endolysosomal dysfunction caused by perturbation of the evolutionarily conserved Retromer complex, highlighting Retromer’s neuroprotective function.

## Introduction

Due to the vast numbers of integral membrane proteins (and their associated proteins and lipids) that require efficient and timely transport through the endo-lysosomal network, the cellular consequences of network dysfunction are widespread. Commonly linked to neurodegenerative diseases through highly complex phenotypes, network dysfunction includes impaired synaptic transmission, accelerated secretion, and reduced lysosomal catabolism associated with the accumulation of damaged organelles and abnormal intracellular protein aggregates.

Retromer is a multiprotein complex that couples with accessory proteins to regulate the sequence-dependent sorting of hundreds of integral membrane proteins through the endo-lysosomal network, protecting them from lysosomal degradation^1,2^. Moreover, Retromer plays a key role in regulating Rab7 nucleotide cycling^3–5^. Retromer deficiency has been observed in Alzheimer’s disease patient brain samples, where its depletion or dysfunction can trigger or accelerate amyloid-β (Aβ) and Tau pathologies^6–10^. Subtle Retromer dysfunction is also associated with disease-causing mutations in Parkinson’s disease^11–16^ and selective deletion of a key Retromer gene in neurons causes an amyotrophic lateral sclerosis-like phenotype in mice^17^.

It is imperative to understand the global cellular consequences of Retromer depletion and/or dysfunction to fully appreciate and contextualise the increasing interest in Retromer as a potential therapeutic target for these diseases. Here, by employing an integrated proteomic approach, we provide an unprecedented view of the impact of Retromer dysfunction on endolysosomal homoeostasis and health.

## Results

### Retromer depletion induces severe morphological changes to the endo-lysosomal network

**Fig. 1:**
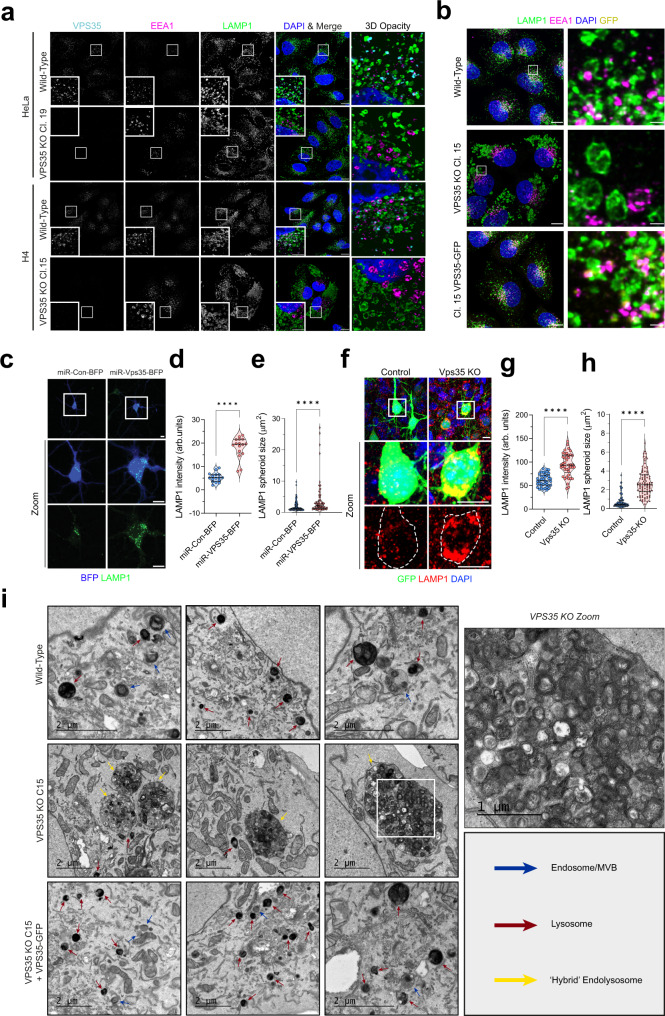
Retromer depletion imposes severe morphological changes on the endolysosomal network. **a** Wild-type and VPS35 KO H4 and HeLa were fixed and immuno-stained for VPS35, EEA1, LAMP1 and DAPI. Scale bars: 20 µm and 5 µm in zoomed panels. Data representative of three experimental repeats. **b** Wild-type, VPS35 KO Cl.15 and Cl.15 + VPS35-GFP H4 cells were fixed and immuno-stained for LAMP1, EEA1 and DAPI. Scale bars: 20 µm and 2 µm in zoomed panels. Data representative of three independent repeats. **c** Cortical neurons, from E18.5 mouse embryos, were cultured and transfected with blue fluorescent protein (BFP) (control) or with miRNA-VPS35-BFP at DIV3 and immune-stained with anti-LAMP1 at DIV7. Scale bar, 10um. **d** Quantification analysis of LAMP1 intensity (arbitrary units) from (**c**). Median and quartiles, *n* = 13 miR-Con-BFP and 16 miR-VPS35-BFP neurons from eight embryos per group, unpaired two-tailed *t* test, *p* < 0.0001. **e** Quantification analysis of LAMP1 spheroid size from c. Median and quartiles, *n* = 136 compartments from 16 miR-Con-BFP neurons and 84 compartments from 16 miR-VPS35-BFP neurons, from eight embryos per group, unpaired two-tailed *t* test *p* < 0.0001. **f** Vps35^f/f^ embryos at E15.5 or E18.5 were in utero electroporated with plasmids of pCAG-GFP (control) or pCAG-Cre-GFP (Vps35^KO^) and their neocortical brain sections at P14 or P30 were examined. Representative Z-stack projection images from P14 brain sections are shown. Scale bar, 10um. **g** Quantification analysis of LAMP1 intensity (arbitrary units) from D. Median and quartiles, *n* = 42 control neurons and 42 Vps35 KO neurons from 12 mice per group. Unpaired two-tailed *t* test, *p* < 0.0001. **h** Quantification analysis of LAMP1 spheroid size from D. Median and quartiles, *n* = 85 compartments from 42 control neurons and 92 compartments from 42 Vps35 KO neurons from 12 mice per group. Unpaired two-tailed *t* test, *p* < 0.0001. In all graphs: blue—control, red—Vps35 KO. **i** Transmission electron micrographs of endolysosomal compartments in cell lines, blue arrows denote endosomes/multivesicular bodies (MVBs), red = lysosomes and yellow = hybrid endo-lysosomes. Scale bars: 2 µm and 1 µm in zoomed panel. Data representative of one experiment.

We generated clonal knockout (KO) H4 neuroglioma cell lines targeting the core *VPS35* component of Retromer, and rescued these lines through stable re-expression of functional VPS35-GFP (Supplementary Fig. 1a)^18^. VPS35 deletion in H4 cells induced profound morphological changes to the endo-lysosomal network, far greater than observed in corresponding VPS35 KO HeLa cells (Fig. 1a, b, Supplementary Fig. 1b)^19^. We therefore pursued the H4 neuroglioma cell model as a tool to understand the role of Retromer in maintaining lysosomal homoeostasis, particularly in the brain.

To validate the physiological relevance of this approach, we addressed whether VPS35 deficiency induces similar morphological changes to the endo-lysosomal network in mouse neurons. Primary neurons (from E18.5 mouse cortex) were cultured and at division day 3 (DIV3) they were transfected with plasmids of miRNA (miR)-Vps35-BFP (to suppress Vps35 expression) or miR-blue fluorescence protein (BFP) (Fig. 1c). At DIV7, the LAMP1-positive compartments were evaluated. The miR-Vps35-BFP suppressed Vps35 expression efficiently^20,21^, revealing that the Vps35-deficient neurons showed increased LAMP1 compartment volume and signal intensity (Fig. 1d, e) and exhibited similar morphological changes as those in H4 neuroglioma cells. We further verified the increase of LAMP1 compartment volume and signal intensity in P14 and P30 mouse cortical neurons in vivo by use of a Vps35^f/f^ mouse model whereby cortical neuronal Vps35 was depleted by *in utero* electroporation (IUE) of a plasmid of CAG promotor driven GFP-Cre (at E15.5 or E18.5) (Fig. 1f–h). These results thus provide in vitro and in vivo evidence for the enlarged LAMP1-positive compartments in Retromer deficient neurons, reflective of the phenotype we observed in H4 neuroglioma cells. These data highlight the suitability of H4 cells to recapitulate the lysosomal disruption observed in primary neuronal culture systems upon Retromer deletion.

In VPS35 KO H4 cells, transmission electron microscopy revealed dramatically enlarged hybrid endo-lysosomal compartments, up to 10 µm in diameter, loaded with undigested membranous intraluminal material (Fig. 1c). These structures are reminiscent of those observed in Alzheimer’s, Parkinson’s and Lewy Body disease patients^22–24^. BSA-gold uptake assays unambiguously defined the enlarged hybrid compartments in VPS35 KO H4 cells as endocytic in nature (Supplementary Figs. 1c, d). The pool of small, dense-core lysosomes observed in wild-type cells was depleted in VPS35 KO cells, suggesting that the swollen VPS35 KO compartments arrive from endosome-lysosome fusion events, followed by subsequent failure to clear the luminal content and reform lysosomes from the hybrid compartment. Importantly, VPS35-GFP re-expression facilitated the clearance of this accumulated material to rescue endo-lysosomal morphology and the population of dense-core lysosomes (Fig. 1c). Epidermal growth factor (EGF) uptake assays confirmed that aberrant VPS35 KO lysosomes exhibited a limited degradative capacity (Supplementary Figs. 1e–g).

### LysoIP proteomics reveals a fingerprint of lysosomal dysfunction in VPS35 KO cells

**Fig. 2:**
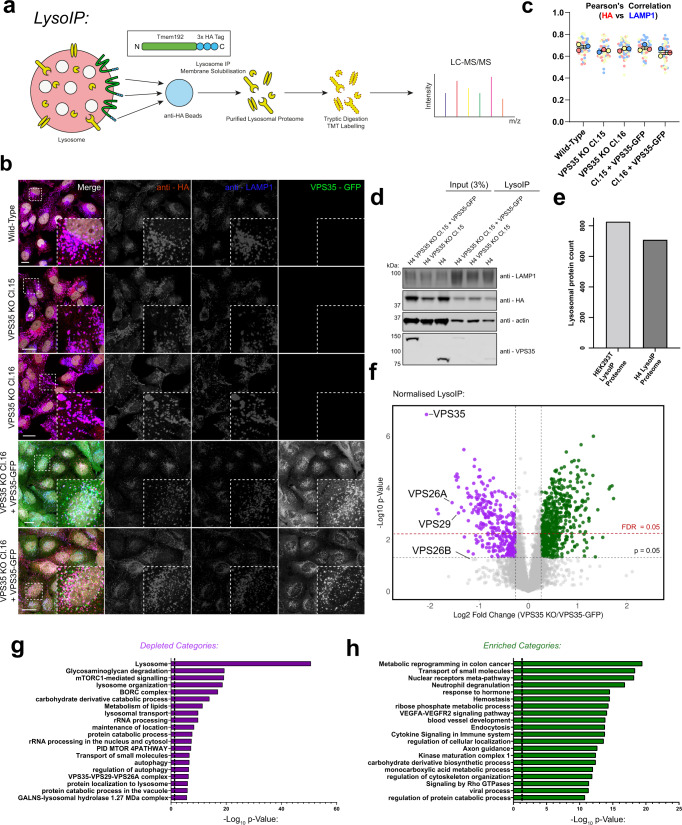
Development of organelle restricted proteomics approach to characterise VPS35 KO lysosomes. **a** Schematic depicting LysoIP methodology coupled to TMT-based quantitative proteomics. **b** TMEM192–x3HA labels lysosomes with a high degree of specificity. Representative confocal images of H4 cell lines transduced to express TMEM192-x3-HA prior to fixation and immuno-staining for HA, LAMP1 and DAPI. Scale bars: 20 µm. **c** TMEM192-x3-HA and LAMP1 signals were quantified by measuring Pearson’s correlation coefficient. *n* = 3 independent experiments, means ± SEM, one-way ANOVA with Dunnett’s multiple comparisons tests. Wild-type vs VPS35 KO Cl.15, VPS35 KO Cl.16, VPS35 KO Cl.15 + VPS35-GFP or VPS35 KO Cl.16 + VPS35-GFP; *p* values = 0.3055, 0.6723, 0.9766 and 0.0924 respectively. Datapoints are coloured by independent repeat. **d** LysoIP efficiently precipitates lysosomes from wild-type, VPS35 KO and VPS35-GFP rescue cell lines. LysoIP was performed on indicated cell lines prior to immuno-blotting with anti-LAMP1 (to assess lysosome enrichment), β-actin, HA and VPS35. kDa = kilodaltons. **e** The lysosomal proteome of H4 exhibits a high degree of overlap with the published proteome of HEK293T-derived lysosomes. **f** A cohort of proteins are significantly relatively enriched (477 proteins) or depleted (246 proteins) from VPS35 KO lysosomes compared to both wild-type and rescue cells. The VPS35 KO/VPS35-GFP abundance ratio is displayed as a volcano plot. Data were normalised relative to total protein count and used to generate a volcano plot from *n* = 7 independent experiments, presented as ratio of Log_2_ VPS35 KO/KO + VPS35 GFP vs −Log10 *p*-value (thresholds set at *p* = 0.05 and fold Log_2_ change ± 0.26) two-tailed paired *t* test. A 5% FDR threshold is overlaid in red, Benjamini–Hochberg correction. Magenta—significantly depleted in VPS35 KO, green—significantly enriched in VPS35 KO. **g**, **h** Cohorts of depleted (**f**) or enriched (**g**) proteins in VPS35 KO lysosomes converge into functional groupings. Gene ontology analyses of significantly enriched or depleted proteins, (log_2_ fold change ± 0.26, *p* < 0.05), describing depleted and enriched functional categories plotted against significance, hypergeometric test.

To unbiasedly define the altered lysosome morphology in VPS35 KO H4 cells we coupled a lysosome immunoprecipitation (LysoIP) methodology with quantitative proteomics (Fig. 2a)^25^. We transduced wild-type, VPS35 KO and VPS35-GFP-expressing H4 cells with transmembrane protein 192 (TMEM192), C-terminally flanked by three tandem HA epitopes (Supplementary Fig. 2a). Immunostaining of transduced cells with anti-HA and LAMP1 antibodies demonstrated the specificity of TMEM192-x3-HA to label lysosomes (Fig. 2b) and homogeneity of labelling across cell lines was confirmed by quantifying colocalisation between the fluorescent signals (Fig. 2c). Immunoblotting of anti-HA LysoIPs showed a strong enrichment for the lysosomal marker LAMP1 that appeared highly consistent between wild-type, VPS35 KO and VPS35-GFP rescue-derived lysosomes (Fig. 2d). From isobaric tandem mass tagging (TMT) and LC-MS/MS quantification we obtained a LysoIP dataset from wild type cells. While the presence of non-specific interactors in the immunoprecipitation protocol cannot be excluded, this dataset was highly enriched for lysosomal proteins (Supplementary Fig. 2b, c), which contained 709 of the 828 proteins previously described as associated with lysosomes in HEK293T cells (Fig. 2e)^25^.

We generated proteomic LysoIP datasets from wild-type, VPS35 KO and rescue cell lines. VPS35 KO H4 cells exhibited a dramatic increase in protein abundances following LysoIP, reflective of the increased lysosomal number and size observed by microscopy, and consistent with the known activation of the master lysosomal biogenesis regulator transcription factor EB (TFEB) upon Retromer dysfunction in various cell types^3,26^ (Supplementary Fig. 3a, Supplementary Data 1). Data were therefore normalised to total peptide amount to provide relative changes to the lysosomal proteome (Fig. 2f, Supplementary Data 2). As expected, VPS35 itself was the top depleted protein in VPS35 KO cells compared to wild-type or VPS35-GFP control samples. Although steps were taken to mitigate the effects of TMT ratio compression, the apparent small 4-fold reduction in VPS35 quantification in this experiment is likely an underestimate resulting from this phenomenon. Comparison of LysoIP proteomes from VPS35 KO H4 cells relative to wild-type and VPS35-GFP rescues revealed significantly depleted (246 proteins) and enriched (477 proteins) proteins (log_2_ fold change ± 0.26, *p* < 0.05). Gene ontology analysis revealed an overall loss in lysosomal identity and an increased abundance of various pathways including metabolic reprogramming and small molecule transport (Fig. 2g, h, Supplementary Data 3). Importantly, a more stringent 5% false discovery rate (FDR) statistical cut-off also revealed a similar profile of enriched and depleted proteins, providing confidence in these results (Fig. 2f, Supplementary Figs. 3b, c).

**Fig. 3:**
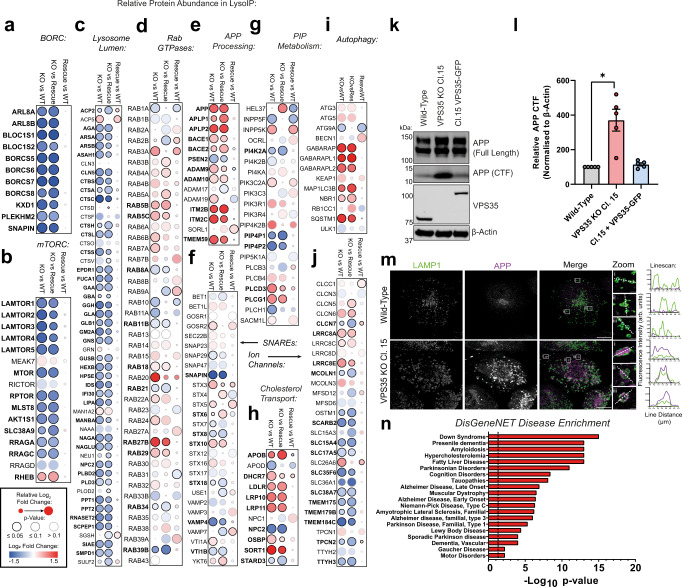
LysoIP proteomics reveals a fingerprint of lysosomal dysfunction in VPS35 KO cells. **a**–**j** A range of functional networks and protein families were relatively depleted or enriched in lysosomes derived from VPS35 KO H4 cells relative to wild-type and VPS35-GFP-expressing rescue cells. Dot-plots representing log_2_ fold change and *p*-value in quantified abundances of (**a**) BORC; (**b**) mTORC1 complexes; (**c**) lysosomal lumen proteins; (**d**) Rab GTPases; (**e**) APP processing proteins; (**f**) vesicle SNAREs; (**g**) PIP metabolism; (**h**) cholesterol transport; (**i**) autophagy; and (**j**) lysosomal solute channels, two-tailed paired *t* tests. Log_2_ fold change, relative change and *p*-value score are depicted by dot colour, size and outline respectively. **k** Proteolytic processing of APP is enhanced in VPS35 KO H4 cells. Cell lysates from the indicated cell lines were immuno-blotted using anti-APP (full length and CTF), VPS35 and β-actin antibodies. APP-CTF levels in VPS35 KO clone were rescued upon re-introduction of VPS35-GFP. kDa = kilodaltons. **l** Quantification of APP-CTF signal intensity (*n* = 5 independent experiments), means ± SEM, one-sample t-tests with Holm–Šídák correction, adjusted *p* = 0.025 (VPS35 KO Cl.15), 0.1655 (Cl.15 + VPS35-GFP). Red—VPS35 KO clone 15, blue—VPS35 Clone 15 + VPS35-GFP. **m** APP accumulates in LAMP1-positive compartments in VPS35 KO cells. Wild-type and VPS35 KO H4 were fixed and immuno-stained for LAMP1 and APP and linescan analysis was used to demonstrate colocalisation (arbitrary units). Scale bar = 20 µm and 1 µm in zoomed panels. Data representative of 3 independent repeats. **n** Proteins enriched in the VPS35 KO LysoIP dataset are associated with neurodegenerative disease. Enrichment of selected DisGeneNET disease categories represented by significantly enriched proteins in the VPS35 KO LysoIP dataset (log_2_ fold change ± 0.26, *p* < 0.05) relative to both wild-type and VPS35-GFP-expressing control conditions, hypergeometric test.

A range of protein–protein interaction networks were significantly depleted and enriched in the VPS35 KO LysoIP dataset (Supplementary Figs. 3d, e). All components of the BORC complex were depleted from VPS35 KO lysosomes and rescued by VPS35-GFP re-expression (Fig. 3a). The BORC complex positions lysosomes by coupling to kinesin-mediated microtubule transport via the adaptor protein Arl8, which was also depleted from VPS35 KO lysosomes^27^. Lysosomal recruitment of BORC is regulated by mTORC1/Ragulator. Indeed, mTOR and associated components including all Ragulator subunits LAMTOR1-5 and RagA/C GTPases were depleted in VPS35 KO lysosomes (Fig. 3b, Supplementary Fig. 3e). Rheb, an amino acid-responsive activator of mTORC1^28^, was the only component of the mTORC1 machinery to be significantly enriched in VPS35 KO lysosomes (Fig. 3b). Quantification of whole cell lysates confirmed that respective levels of mTOR and Raptor were unchanged as result of VPS35 deletion (Supplementary Fig. 4a–c), although their levels were diminished in LysoIP immunoprecipitates (Supplementary Fig. 4d). To further confirm this observation, we investigated the ability of mTORC1 to translocate to lysosomal membranes following amino acid starvation and re-feeding. In VPS35 KO cells, mTOR and RagC demonstrated a reduced colocalisation with LAMP1 under these conditions (Supplementary Figs. 4e–h).

A wide cohort of luminal proteins, a large proportion of which are hydrolytic enzymes, were significantly depleted in VPS35 KO cells, including proteases, lipases, nucleases, and glycosidases including β-galactosidase (GLB1), which has previously shown to exhibit reduced activity in Retromer-depleted HeLa cells^29^ (Fig. 3c). Notably, the lysosomal acid glucosylceramidase GBA, which is genetically linked to Gaucher’s disease and Parkinson’s disease, was significantly depleted in VPS35 KO lysosomes^30^, as were a wider cohort of enzymes associated with lysosomal storage diseases^31^.

Rab GTPases are crucial regulators of membrane identity and transport^32^. Rab5a and Rab5b were significantly enriched in lysosomes from VPS35 KO H4 cells, reflective of increased mixing between early and late endosomes and defective resolution of these compartments (Fig. 3d). Rab7a was also significantly enriched in VPS35 KO cells compared to wild-type controls, as was Rab29, with the strongest enrichment being Rab27b, a late endosomal GTPase that regulates lysosomal exocytosis. Rab GTPases involved in late endosomal fusion with phagosomes and autophagosomes were dysregulated, including Rab20, Rab21 and Rab34^33–35^. Rab39b, which has been reported to regulate alpha-synuclein accumulation and has loss-of-function mutations in X-linked Parkinson’s disease, was strongly depleted in VPS35 KO lysosomes^36,37^ (Fig. 3d).

Within the data we also identified: an enrichment of proteins involved in APP processing and metabolism, including an accumulation of APP in VPS35 KO lysosomes (Fig. 3e); depletion of the late endosomal SNARE proteins STX8 and VTI1B (Fig. 3f); dysregulation of phosphatidylinositol-4-phosphate (PI(4)P) metabolism, including depletion of PIP4P1 (TMEM55B), a regulator of perinuclear lysosomal transport and v-ATPase assembly^38,39^ (Fig. 3g); perturbation of cholesterol influx and egress from lysosomes, which has been linked strongly with late onset, atypical cognitive decline^40^ (Fig. 3h); enrichment of autophagy markers (Fig. 3i); and alterations to lysosomal solute channels that regulate transmembrane transport to maintain lysosomal homoeostasis, including CLCN7, a key Cl^-^/H^+^ antiporter that balances charge within the lysosomal lumen^41^, and TMEM175, recently linked to Parkinson’s disease^42^ (Fig. 3j). Taken together, these data highlight the scale of lysosomal dysfunction induced by Retromer depletion.

We focussed on the enrichment of APP, which shuttles between the *trans*-Golgi network (TGN), endosomal network and plasma membrane under steady-state conditions and can undergo multiple proteolytic cleavage steps performed by α-, β-, and γ-secretase enzymes^43^. APP mutations that alter its proteolytic processing are causally linked to Alzheimer’s disease through the generation of neurotoxic amyloid aggregates^43^. We noticed that VPS35 KO H4 cells demonstrate an abnormal accumulation of cleaved APP in the whole cell lysate, measured as a low molecular weight C-terminal fragment resulting from α- or β-secretase cleavage events (Fig. 3k–l). A similar accumulation of APP CTFs was recently reported in Vps35-depleted mouse neurons^44^. Levels of full-length APP were unaffected (Supplementary Fig. 4i). In wild-type and VPS35-GFP-expressing cells, APP predominantly localises within the perinuclear region, mainly colocalising with TGN46 and displaying minimal overlap with LAMP1 (Supplementary Fig. 4j). In contrast, VPS35 KO cells demonstrated a striking accumulation of APP on the limiting membrane or within LAMP1-positive compartments (Fig. 3m and Supplementary Fig. 4j). APP misprocessing and accumulation within the endo-lysosomal network is a crucial hallmark of Alzheimer’s disease, and is sufficient to induce lysosomal dysfunction^45^. Interestingly, DisGeneNet categories associated with significantly enriched proteins on VPS35 KO lysosomes included ‘Down Syndrome’, ‘Presenile Dementia’, ‘Amyloidosis’, ‘Alzheimer Disease’, ‘Parkinson Disease’, amongst others, indicating that the proteomic changes observed in these cells correlate with established phenotypes of neurodegeneration (Fig. 3n, Supplementary Fig. 4k, Supplementary Data 4).

### Correlative proteomic analyses reveal a signature of lysosomal exocytosis in VPS35 KO cells

**Fig. 4:**
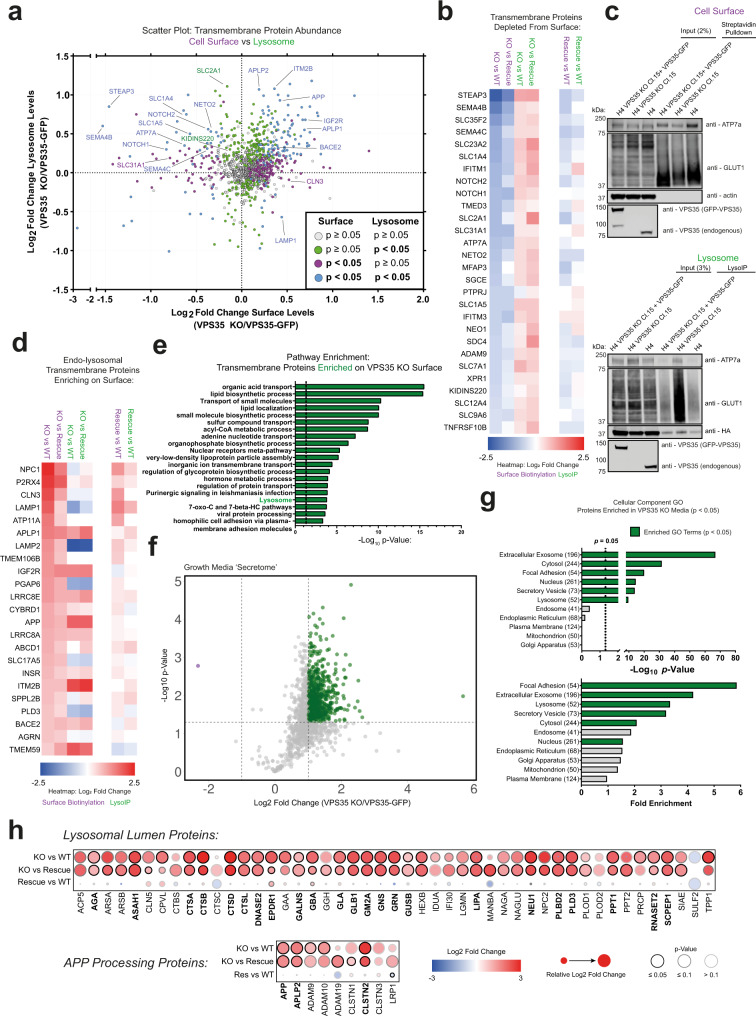
The cell surface proteome is re-modelled in VPS35 KO H4 cells. **a** Scatter plot of VPS35 KO/VPS35-GFP transmembrane protein abundances in the cell surface proteome (*x*-axis) versus the LysoIP proteome (*y*-axis). Datapoints coloured based on *p*-value scores in each experiment (green—significantly altered in LysoIP, magenta—significantly altered in surface proteome, blue—significantly altered in LysoIP and surface proteome), derived from two-tailed paired *t* tests. **b** Heatmap depicting the loss of transmembrane proteins from the cell surface proteome (purple columns) with concomitant enrichment in the LysoIP proteome (green columns) in VPS35 KO cells relative to wild-type and VPS35-GFP rescue controls. Proteins coloured by Log_2_ fold change between VPS35 KO and wild-type (WT) or VPS35-GFP (rescue) samples. **c** Cell surface and lysosomal proteomes were subjected to immuno-blotting with antibodies recognising defined Retromer cargoes (ATP7a and GLUT1). Data representative of 6 (cell surface) and 7 (LysoIP) independent repeats. kDa = kilodaltons. **d** Heatmap depicting the enrichment of transmembrane proteins in the cell surface proteome (purple columns) and corresponding abundance in the LysoIP proteome (green columns) in VPS35 KO cells relative to wild-type and VPS35-GFP rescue controls. Proteins coloured by Log_2_ fold change between VPS35 KO and wild-type (WT) or VPS35-GFP (rescue) samples. **e** Analysis of significantly enriched pathways in the VPS35 KO transmembrane cell surface proteome relative to wild-type and VPS35-GFP rescue controls, hypergeometric test. **f** Volcano plot of VPS35 KO/VPS35-GFP protein abundances in the growth media ‘secretome’. 325 proteins were significantly enriched in the growth media of VPS35 KO cells compared to both wild-type and rescue sample (Log_2_ fold change > 1, *p* < 0.05), two-tailed paired *t* tests. Magenta—significantly depleted in VPS35 KO, green—significantly enriched in VPS35 KO. **g** Gene ontology analysis of cellular components enriched in the VPS35 KO ‘secretome’. Statistically significant categories (*p* < 0.05) displayed in green, Fisher’s exact test. **h** Dot-plot predicting lysosomal luminal protein and APP processing protein abundances in the VPS35 KO ‘secretome’ relative to wild-type and VPS35-GFP rescue control samples. Log_2_ fold change, relative change and *p*-value score are depicted by dot colour, size and outline respectively, two-tailed paired *t* tests.

Retromer dysfunction induces a widespread reduction in the cell surface expression of integral membrane proteins as they become sequestered within internal endolysosomal compartments^46^. By coupling surface-restricted biotinylation and LysoIP methodologies with quantitative proteomics, we attained a global, unbiased overview of how Retromer depletion triggered shifts in integral membrane protein abundances at the cell surface and lysosome, respectively (Fig. 4a, Supplementary Figs. 5a, b, Supplementary Data 2). We observed a cohort of integral membrane proteins that were depleted from the cell surface and became enriched in the lysosome in VPS35 KO cells, including known Retromer cargoes such as GLUT1 (SLC2A1), STEAP3, SEMA4B and SEMA4C, NOTCH1 and NOTCH2, NETO2, KIDINS220, the neutral amino acid transporters SLC1A4 and SLC1A5, and the copper transporters ATP7A and SLC31A1^46–49^ (Fig. 4b). We validated GLUT1 and ATP7A re-routing from the cell surface into TMEM192-3xHA-positive lysosomes by Western blotting and immunostaining (Fig. 4c, Supplementary Fig. 5c). A number of these cargoes, for example SEMA4B/C, NOTCH1/2, NETO2 and KIDINS220, regulate essential neuronal pathways such as axonal guidance^50,51^, and synaptic transmission^52–54^, and are associated with neuronal disease and disorders^55,56^. These data underscore the protective role of Retromer in regulating the integrity of the plasma membrane proteome alongside maintaining lysosomal function.

Interestingly, a cohort of lysosomal integral membrane proteins became enriched on the cell surface in VPS35 KO H4 cells, including the lysosomal glycocalyx components LAMP1 and LAMP2, the cholesterol transporter NPC1, the Juvenile Neuronal Ceroid Lipofuscinosis associated CLN3, CI-MPR (IGF2R), and a cluster of APP processing-related proteins including APP, the β-secretase BACE2, the APP-like protein and β-secretase substrates APLP1 and APLP2^57^, and APP-binding protein ITM2B^58,59^ (Fig. 4d). Specifically, the C-terminal fragment of APP was found to enrich at the cell surface in VPS35 KO cells (Supplementary Fig. 5d). Gene ontology analysis reflected these changes, including depletion of pathways related to cell morphogenesis, adhesion, synapse organisation and transmembrane transport (Supplementary Fig. 5e), which correlate with enrichment in the lysosome (Supplementary Fig. 5f), and a concomitant increase in metabolic pathways and lysosomal proteins at the cell surface, amongst others (Fig. 4e, Supplementary Data 3).

Enrichment of lysosomal proteins at the cell surface could reflect increased lysosomal exocytosis, a process considered to be compensatory for lysosomal stress that may mediate cell-to-cell transfer of pathogenic aggregates such as α-synuclein and APP fragments^60–63^. A cell culture ‘secretome’ revealed a dramatic increase in proteins in the growth media of VPS35 KO H4 cells that was rescued by VPS35-GFP re-expression (Fig. 4f, Supplementary Data 1). Gene ontology analysis revealed a significant enrichment of ‘focal adhesion’, ‘extracellular exosome’, ‘lysosome’ and ‘secretory vesicle’ cellular component categories (Fig. 4g, Supplementary Data 5). While the contribution of cell death to this extracellular protein content cannot be excluded, the predominant enrichment of lysosomal and exosomal content may be indicative of lysosomal exocytosis. Moreover, autophagic cargo receptor Sequestome-1 (SQSTM1) was prominently enriched in the secretome of VPS35 KO cells, suggestive of the release of autophagic material (Supplementary Data 1). A wide cohort of luminal lysosomal proteins, which were relatively depleted in the VPS35 KO LysoIP dataset (Fig. 3c), were significantly enriched in the VPS35 KO secretome (Fig. 4h). Among these, some have been reported to undergo secretion from the biosynthetic pathway upon Retromer depletion^64–66^. However, we observed that CTSD colocalised completely with LAMP1 and displayed no evidence of accumulation within the biosynthetic pathway (Supplementary Fig. 5g). We therefore posit that in addition to biosynthetic pathway ‘leakage’, the delivery of lysosomal enzymes can be maintained in VPS35 KO cells, and their extracellular release may arise from lysosomal exocytosis, with CTSD being predominantly detected in a precursor state due to ineffective pH-dependent proteolytic activation in the endo-lysosomal network.

We also noticed an enrichment of APP in the secretome of VPS35 KO cells, alongside related substrates of the β- and γ-secretase enzymes APLP2 and CLSTN2, respectively^57,67^ (Fig. 4h). Impaired APP processing and increased extracellular release of APP and Aβ fragments have been reported upon VPS35 depletion^9,68^. Moreover, APLP2 was the most significantly enriched protein in the cerebrospinal fluid (CSF) of neuronal Vps35 KO mice, and in human Alzheimer’s disease patients^10^. APP enrichment within the VPS35 KO secretome may therefore be indicative of altered amyloidogenic processing and release from the cell surface.

Taken together, these data indicate an increased release of lysosomal contents from the cell surface in response to the profound lysosomal stress observed in VPS35 KO H4 cells. This emphasises the neuroprotective role of Retromer in safeguarding against lysosomal dysfunction and extracellular release of cytotoxic contents such as APP cleavage products. The correlation of these findings with mouse and human CSF data highlights the possibility that the detection of APP, APLP2 or other components of the VPS35 KO secretome could be diagnostic tools for identifying lysosomal dysfunction in cellular and model organism systems^10^.

### RNA sequencing of VPS35 KO H4 cells reveals transcriptional reconfigurations

Retromer dysfunction is known to induce TFEB activation^3,26^. To directly investigate whether changes in the lysosomal proteome in VPS35 KO H4 cells were due to a coordinated transcriptional response through, for example, the coordinated lysosomal expression and regulation (CLEAR) network, we performed RNA sequencing (RNA-seq) of wild type, VPS35 KO and VPS35-GFP H4 cells (Supplementary Figs. 6a, b, Supplementary Data 6). Examination of RNA abundances of the lysosomal CLEAR network genes documented in HeLa cells^69^ revealed significant upregulation of *ACP5*, *ASAH1*, *CTSB*, *CTSD*, *CTSS*, *GAA*, *NPC2*, *PSAP* and *TPP1* (Supplementary Fig. 6c, Supplementary Data 7). Next, we utilised gene set enrichment analysis (GSEA) to characterise up- and downregulated pathways in VPS35 KO cells (Supplementary Data 8). This revealed enrichment of cellular component genes including endo-lysosomal genes, most notably subunits of the v-ATPase, as well as components of the mitochondrial respiratory chain complex; many of which are known TFEB target genes^70–73^ (Supplementary Fig. 6d). Moreover, there was a significant enrichment of genes associated with the KEGG pathways of Parkinson’s, Alzheimer’s, and Huntington’s diseases (Supplementary Figs. 6e–g).

**Fig. 5:**
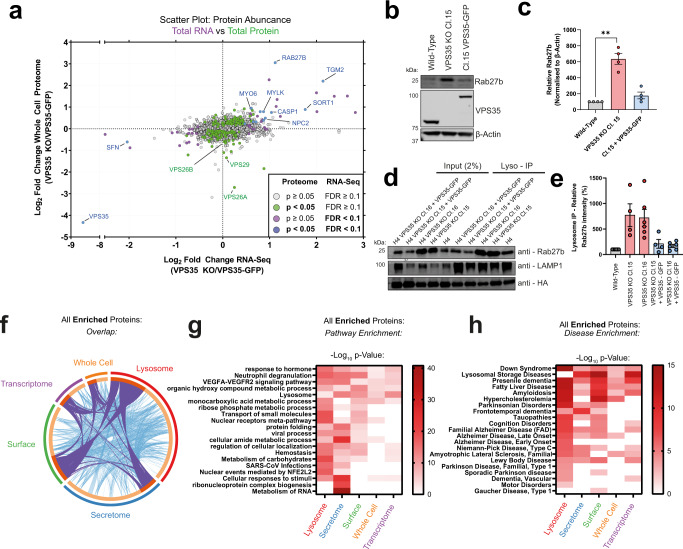
Correlative mapping of ‘omic’ datasets reveals transcriptional upregulation of Rab27b and enriched diseases. **a** Correlative analysis between RNA-Seq and the whole cell proteome reveals upregulation of a specific cohort of proteins. Scatter plot of VPS35 KO/VPS35-GFP RNA-Seq transcript abundances (*x*-axis) versus the protein abundances in the whole cell proteome (*y*-axis). Datapoints are coloured based on *p*-value scores in each experiment (green—significantly altered in whole cell proteome, magenta—significantly altered in RNA-Seq, blue—significantly altered in whole cell proteome and RNA-Seq), derived from two-tailed paired *t* tests. **b**–**e** Western blot and quantification of Rab27b protein levels in the whole cell lysates (**b**, **c**) and lysosomes (**d**, **e**) of wild-type, VPS35 KO, or VPS35-GFP rescue expression lines. Means ± SEM, one-sample two-tailed *t* tests with Holm–Šídák correction. For (**c**), adjusted *p* = 0.0082 (VPS35 KO Cl.15), 0.1603 (Cl.15 + VPS35-GFP). *n* = 4 independent repeats. For (**e**) *p* = 0.162 (VPS35 KO Cl.15), 0.0537 (VPS35 KO Cl.16), 0.2932 (VPS35 KO Cl.15 + VPS35-GFP), 0.162 (VPS35 KO Cl.16 + VPS35-GFP), data representative of 6 (wild type, VPS35 KO Cl.16 and VPS35 KO Cl.16 + VPS35 −GFP) and 4 (VPS35 KO Cl.15 and VPS35 KO Cl.15 + VPS35-GFP) independent repeats. kDa = kilodaltons. **f** Circos plot of overlap of significantly enriched proteins in VPS35 KO samples compared to both wild-type and VPS35-GFP-expressing rescue cells across all datasets. **g**, **h** Pathway enrichment meta-analysis of significantly enriched categories (**g**) and associated DisGeneNET categories (**h**) in VPS35 KO cells compared to both wild-type and VPS35-GFP-expressing rescue cells across all datasets, hypergeometric test. Datasets coloured to represent proteins significantly enriched in VPS35 KO: green—surface proteome, magenta—RNA-Seq transcriptome, orange—whole cell proteome, red—LysoIP proteome, blue—growth media ‘secretome’ proteome.

To refine our RNA-seq experiments, we performed whole-cell TMT proteomics of wild-type, VPS35 KO and VPS35-GFP rescue H4 samples (Supplementary Fig. 7a–c, Supplementary Data 1–2). We correlated RNA transcript abundances with protein abundances to identify proteins that were upregulated at the transcriptional and proteomic level (Fig. 5a). Included within this cohort were *Rab27b*, *SORT1* and *NPC2*, which were also significantly perturbed in the VPS35 KO lysosomal proteome (Fig. 3c, d, h). The endosomal sorting of the lysosomal hydrolase receptor SORT1 is Retromer dependent^29,74,75^, and transcriptional upregulation may therefore reflect a compensatory mechanism to supply lysosomes with hydrolases in response to SORT1 mistrafficking and lysosomal dysfunction. Similarly, NPC2 upregulation may constitute a mechanism to restore cholesterol metabolism and egress from lysosomes due to perturbed localisation of NPC2 (Fig. 3h). Moreover, correlation of LysoIP protein abundances with RNA-Seq data confirmed many of these insights, while demonstrating that despite their transcriptional upregulation a cohort of proteins including NPC2, CTSD, CPQ, HEXB, GAA and LAMTOR4 are conversely depleted from VPS35 KO lysosomes (Supplementary Fig. 7d).

Rab27b was the most abundantly enriched protein in the VPS35 KO total cell proteome and was concomitantly enriched at the transcriptional level—this GTPase was also enriched in the lysosomal proteome (Figs. 3d and 5a). Rab27b regulates translocation of late endosomes to the cell periphery and their exocytosis^76^, and was recently shown to be transcriptionally regulated by the cytoprotective transcription factor Nrf2^77^. In Parkinson’s disease, higher expression of Rab27b has been reported in patient brain samples^78^, where it may promote the cell-to-cell transmission of pathogenic alpha-synuclein aggregates through a lysosomal exocytosis and re-uptake mechanism^78,79^. We validated the significant increase and rescue of Rab27b in VPS35 KO cells by Western blotting of whole cell lysate, and observed enrichment on LysoIP immunoprecipitates, though this effect was not statistically significant (Fig. 5b–e). Given the enrichment of lysosomal proteins, including APP, in the secretome of VPS35 KO cells, we speculate that the upregulation of Rab27b observed upon Retromer dysfunction may constitute a transcriptional link to stimulate lysosomal exocytosis.

Meta-analysis of our multi-omic datasets revealed disease signatures including ‘Down Syndrome’, ‘Presenile Dementia’, ‘Amyloidosis’ and ‘Fatty Liver Disease’, which were unanimously enriched across all experimental approaches; and ‘Parkinsonian Disorders’, ‘Alzheimer Disease’, ‘Lewy Body Disease’, ‘Amyotrophic Lateral Sclerosis’ and ‘Niemann-Pick Disease’, among others, which were significantly enriched in multiple datasets (Fig. 5f-h, Supplementary Data 4).

### Retromer recruitment of effector proteins govern lysosomal homoeostasis

**Fig. 6:**
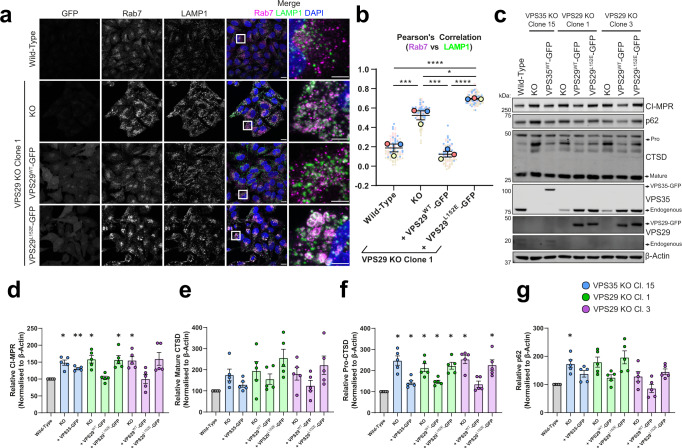
VPS29 KO H4 cells display a hyper-lysosomal recruitment of Rab7. **a** Deletion of VPS29 induces hyper-recruitment of Rab7 to lysosomes, which is rescued by re-expression VPS29^WT^-GFP but not VPS29^L215E^-GFP. Cell lines were fixed and immuno-stained for Rab7, LAMP1 and DAPI. Scale Bars: 20 µm and 5 µm in zoomed panels. **b** Rab7 labelling of lysosomes was quantified by measuring the Pearson’s correlation coefficient between respective fluorescent signals over an *n* of 3 independent experiments. Means ± SEM, one-way ANOVA with Tukey’s multiple comparisons tests. Wild type vs VPS29 KO *p* = 0.0005, wild type vs VPS29^WT^-GFP *p* = 0.5651, wild type vs VPS29^L152E^-GFP *p* < 0.0001, VPS29 KO vs VPS29^WT^-GFP *p* = 0.0001, VPS29 KO vs VPS29^L152E^-GFP *p* = 0.308, VPS29^WT^-GFP vs VPS29^L152E^-GFP *p* < 0.0001. Datapoints coloured by independent repeat. **c**–**g** Destabilisation of the Retromer trimer by VPS35 or VPS29 KO causes an increase in whole cell protein levels of CI-MPR, p62 and pro-CTSD. **c** Representative immunoblot of endogenous protein levels derived from indicated cell lines. **d**–**g** Endogenous levels of indicated proteins were quantified relative to β-actin over *n* = 5 independent experiments. Means ± SEM, one-sample two-tailed *t* tests with Holm–Šídák correction. **d** adjusted *p*-values = 0.0229, 0.0032, 0.0366, 0.8032, 0.0389, 0.0389, 0.9931, 0.1 (**e**) adjusted *p*-values *p* = 0.297, 0.297, 0.297, 0.297, 0.1388, 0.297, 0.3651 (**f**) adjusted *p*-values = 0.0155, 0.0288, 0.0159, 0.0159, 0.0111, 0.0111, 0.07, 0.0206 (**g**) adjusted *p*-values = 0.0478, 0.1242, 0.0771, 0.2008, 0.0771, 0.3049, 0.3899, 0.0771. Blue—VPS35 KO Clone 15, green—VPS29 KO Clone 1, magenta—VPS29 KO clone 3. kDa = kilodaltons.

Finally, we returned to the LysoIP proteomics to seek insight into the mechanism(s) behind the swollen lysosomal phenotype. A possible origin for the multivariate phenotype observed in our datasets is the dysregulation of Rab7 due to impaired recruitment of the Retromer effector and Rab7 GAP TBC1D5^3–5^. Indeed, we observed a hyper-recruitment of Rab7 to LAMP1-positive membranes in VPS35 KO cells, in agreement with observations in HeLa cells from the Steinberg lab^3,4^ (Supplementary Fig. 8a). TBC1D5 is recruited to Retromer though binding to VPS29^80^, which is impaired in the VPS29 L152E mutant^4^. We generated VPS29 KO H4 cells, which exhibited a comparable endo-lysosomal swelling and Rab7 hyper-recruitment to VPS35 KO cells, a phenotype rescued by re-expression of VPS29^WT^-GFP but not VPS29^L152E^-GFP (Fig. 6a–c). VPS35-GFP and VPS29-GFP rescued the accumulation of CI-MPR, but VPS29^L152E^-GFP failed to do so, with similar trends observed for CTSD and p62 (Fig. 6d–g). Importantly, VPS35 protein levels were rescued in VPS29 KOs with VPS29^L152E^-GFP expression, indicating that Retromer assembly was unperturbed, thereby highlighting the importance of TBC1D5 recruitment mediated through VPS29 (Supplementary Fig. 8b). The binding of other VPS29 effector proteins, such as the VAMP7-interacting protein VARP, are also affected by the VPS29 L152E mutation^81^. We therefore cannot exclude additional consequences of this mutation, such as perturbed VAMP7-mediated fusion dynamics, contributing to lysosomal dysfunction. Taken together, these data establish a broad endo-lysosomal phenotype in Retromer-depleted H4 cells that arise, in part, from dysregulation of Rab7 nucleotide cycling.

### VPS35 KO cells fail to efficiently undergo lysosomal reformation events

While the hyper-recruitment and activation of Rab7 likely promotes the rate of late endosome and autophagosome fusion with lysosomes, the decreased lysosomal enrichment of mTORC1, BORC, and PI(4)P metabolising enzymes is diagnostic for a defect in autophagic lysosomal reformation (ALR), membrane tubulation events that resolve lysosomes back to their original size and density^82–84^. Amino acid starvation of wild-type and VPS35-GFP-expressing H4 cells induced a rapid dissociation of mTOR from lysosomes, which was recovered upon re-feeding in amino acid-replete DMEM (Supplementary Fig. 9). In VPS35 KO H4 cells, mTOR was already dissociated from lysosomes prior to amino acid starvation and failed to relocalise to lysosomes upon re-feeding (Supplementary Fig. 9), as shown recently in HeLa cells^3^.

**Fig. 7:**
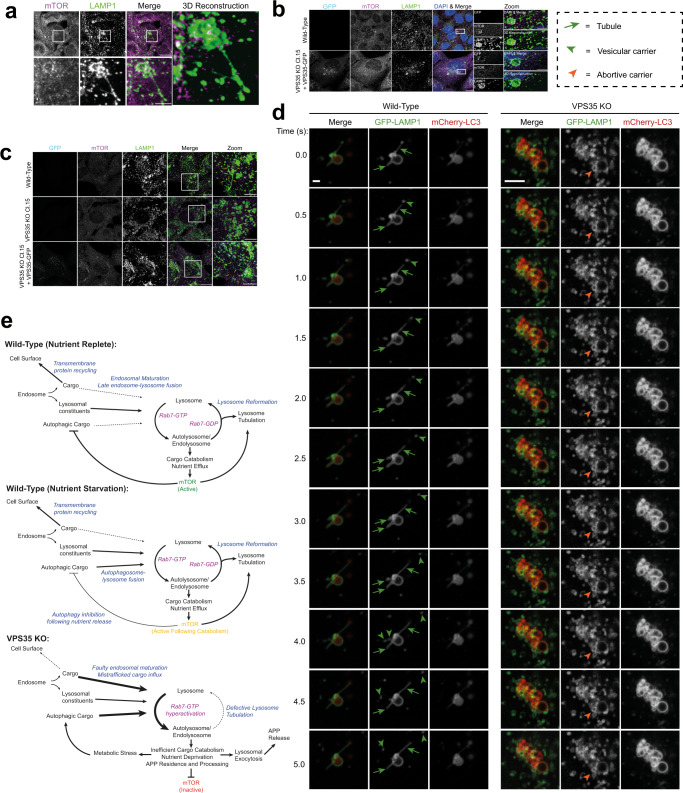
Perturbed autolysosome reformation induces defects to endo-lysosomal morphology in VPS35 KO. **a** Tubular resolution of H4 autolysosomes in response to amino acid starvation. H4 cells were depleted of amino acids for 2 h prior to fixation and immuno-staining for mTOR and LAMP1. Magnified and 3D reconstructed panels depict mTOR and LAMP1 positive tubules emanating from autolysosomes. Scale Bars: 20 µm and 5 µm in zoomed panels. Data representative of 3 independent repeats. **b** Starvation induced autolysosome reformation in H4 is recapitulated in VPS35-GFP cells. H4 and VPS35-GFP cells were depleted of amino acids for 2 h prior to fixation and immuno-staining for DAPI, mTOR and LAMP1. Magnified panels depict mTOR and LAMP1 decorated tubules emanating from autolysosomes co-labelled for VPS35-GFP. Scale Bars: 20 µm and 5 µm in zoomed panels. Data representative of 3 independent repeats. **c** Defective autolysosome resolution in VPS35 KO H4 cells. Cells were depleted of amino acids for 2 h and refed in full media for 15 min prior to fixation and immuno-staining for mTOR and LAMP1. Zoomed panels show mTOR and LAMP1 tubules in H4 and VPS35 GFP compared to the LAMP1 positive, mTOR negative vesicular structures observed in VPS35 KO. Scale Bars: 20 µm and 5 µm in zoomed panels. Data representative of 3 independent repeats. **d** Representative time courses from live cell imaging of LAMP1 positive tubule formation and scission comparing events observed in wild-type and VPS35 KO cells. S = seconds. Data representative of 3 independent repeats. **e** Schematic depicting Rab7/mTOR-dependent activation of lysosome reformation under nutrient replete and starved conditions in wild-type cells. In VPS35 KO, reduced cargo recycling to the cell surface and increased flux into lysosomes, Rab7 hyperactivation, mTOR inactivation and perturbed lysosome tubulation illicit perturbed morphology and function of the endolysosomal network.

Following periods of amino acid starvation extending beyond 60 min, we observed examples of ALR, defined by mTOR recruitment onto the vacuolar portion of LAMP1-positive compartments with extended tubular structures (Fig. 7a). This phenomenon was observed in wild-type and VPS35-GFP-expressing cells during starvation and after amino acid re-feeding (Fig. 7b, c) but was not observed in VPS35 KO cells (Fig. 7c). We expressed GFP-RFP-LC3, a dual-colour autophagic flux reporter that loses GFP fluorescence within acidified autolysosomes. Expression of this reporter revealed extensive tubular networks of branches emanating from LAMP1-positive autolysosomes in wild-type cells upon amino acid starvation or re-feeding (Supplementary Fig. 10). Similar tubular events were far rarer in VPS35 KO cells, indicative of faulty ALR in the absence of mTOR-dependent nutrient sensing.

Live cell imaging revealed dynamic auto-lysosomal tubulation and fission events in wild-type cells expressing LAMP1-GFP and mCherry-LC3, which effectively serve to maintain compartment volume (Fig. 7d, Supplementary Movies 1–6). Auto-lysosome membrane tubulation events, defined by tubulation of a LAMP1-GFP- and mCherry-LC3-positive compartment, were occasionally observed in VPS35 KO cells, but these were less frequent and less likely to undergo productive scission (Fig. 7d, Supplementary Movies 7–12). Taken together, our data therefore reveal a defect in ALR as a major contributing factor in the swollen lysosomal phenotype observed in VPS35 KO cells. Our integrated proteomics approach reveals the dysregulation of several proteins which may contribute to this phenotype, including mTORC1, BORC and PI(4)P-metabolising enzymes.

## Discussion

### Towards a global understanding of the neuroprotective role of retromer

Retromer has been associated with neurodegenerative disease since observation of reduced expression in Alzheimer’s disease patients and the identification of familial causative Parkinson’s disease mutations within the complex^11–16^. Our ‘omics-based approach has provided an unbiased analysis to highlight the detailed role of Retromer in safeguarding the functionality of the cell surface and maintaining lysosomal health. We recently developed an acute knocksideways system for the inactivation of Retromer in HeLa and H4 cells, which directly induces cargo mistrafficking within an hour^18^. In contrast, the extent and severity of the lysosomal phenotype characterised in this manuscript reflects a chronic, “end-point” phenotype that arises over hours to days from a culmination of perturbed cargo sorting, and dysfunctional endosomal maturation, lysosomal catabolism and resolution, with several indirect downstream consequences.

With the emerging concept that small molecule compounds can be used to stabilise Retromer to enhance its neuroprotective function^85,86^, our comprehensive cellular and molecular phenotyping of VPS35 KO H4 cells establishes a roadmap for future translational work to better understand the pathogenic link between Retromer-dependent regulation of lysosomal homoeostasis and individual neurodegenerative diseases, and to inform diagnostic and therapeutic strategies.

### Dysfunctional lysosomal homoeostasis in the absence of retromer

Lysosomal fusion and reformation dynamics are tightly regulated and essential for timely responsiveness to nutrient availability and catabolism. Following fusion of lysosomes with incoming endosomal or autophagic compartments, the resulting hybrid organelles (termed endo-lysosomes and auto-lysosomes, respectively) undergo a reformation process, whereby intraluminal contents are efficiently degraded, catabolites are exported out of the lysosome, and the resulting excess membrane and associated proteins are recycled through a tubulation process that is dependent on connectivity to the cytoskeleton. Through this mechanism, lysosomal size and number are controlled to facilitate cyclical rounds of fusion and reformation over the lifetime of a cell^87,88^. Here, we demonstrate that besides its known role in autophagic flux^4,6,89^, Retromer is required for the ALR pathway to reform lysosomes following productive autophagic fusion events, a key step within the complex pathways that together maintain lysosomal homoeostasis.

Following productive biogenesis of autophagosomes and lysosomal fusion, the delivered contents are efficiently catabolised, resulting in the efflux of nutrients. This is sensed by mTORC1, inducing a reactivation of mTORC1 activity to inhibit further autophagy and stimulate ALR^82^. Loss of mTOR association with lysosomes in VPS35 KO cells, and the mTORC1 depletion observed from LysoIP proteomics, suggests a perturbation to the lysosomal nutrient sensing system. Depletion of lysosomal solute transporters required for nutrient efflux, such as the L-glutamine transporter SLC38A7, may exacerbate this problem in catabolite transport and nutrient sensing at the lysosome (Fig. 3j).

mTORC1 signalling is required to recruit the BORC complex, a key regulator of lysosomal positioning and size^27,90,91^, and BORC is required for the generation of lysosomal tubulation during ALR^83^. Dynamic interconversion between PI(4)P and PI(4,5)P_2_ also contributes to the ALR tubulation process^84,92–94^. Rab7-GTP hydrolysis is crucial for lysosomal reformation, since expression of constitutively active Rab7 or treatment with a non-hydrolysable GTP analogue inhibits ALR^82^. The decrease in PI4K2A, PIP4P1 and PIP4P2, and BORC components from VPS35 KO lysosomes, along with hyper-recruitment of active Rab7, provide mechanistic insight into the observed decreased induction of ALR.

We therefore propose the following working model that underpins the lysosomal swelling phenotype observed in Retromer dysfunctional cells (Fig. 7e). Increased autophagic cargo influx and endosomal cargo missorting from the cell surface, combined with dysregulated endosomal maturation lead to inefficient degradation of lysosomal cargoes. Intraluminal contents accumulate within this environment, including APP. The impaired catabolism of these cargoes prevents effective nutrient efflux into the cytosol, which is required to initiate mTORC1- and BORC-dependent lysosome reformation events. Iterative rounds of fusion events and cargo delivery without effective resolution ultimately compound these problems, leading to the striking enlargement of hybrid organelles seen by light and electron microscopy. Endo- and auto-lysosomal exocytosis may constitute a cytoprotective response that releases undegraded contents from the cell, which in turn may facilitate cell-to-cell spreading of toxic contents such as pathogenic protein aggregates, which is emerging as a defining feature of neurodegenerative diseases^95^. Given that this broad lysosomal phenotype is observed in many models of both Retromer-dependent and Retromer-independent neurodegeneration, our data provide new insights into the neuroprotective role of Retromer in safeguarding lysosomal health.

### Lysosomal exocytosis as a compensatory mechanism in VPS35 KO H4 cells

The enrichment of soluble and transmembrane lysosomal proteins within the VPS35 KO ‘secretome’ is suggestive of lysosomal exocytosis. Retromer suppression has been associated with increased release of Aβ through exosomes in cell culture models^9^. Mass spectrometry of cerebrospinal fluid also recently revealed increased abundance of Tau in VPS35 KO mice and Alzheimer’s disease patient samples compared to controls^10^. In a recent study, Vps35 depleted *Drosophila* demonstrated APP accumulation in presynaptic neurons of the neuromuscular junction, and increased APP levels in extracellular postsynaptic vesicles^68^.

Lysosomal exocytosis has been proposed as a compensatory mechanism in response to lysosomal dysfunction^95^. For example, chemical perturbation of lysosomal homoeostasis with ammonium chloride or bafilomycin A1 in SH-SY5Y cells leads to increased α-synuclein exocytosis and paracrine transfer to neighbouring cells^61^. This pathway may be particularly beneficial to neurons, whereby extracellular release of undegraded lysosomal contents alleviates lysosomal stress, and the released material can be internalised and degraded by neighbouring microglial cells in the brain. While this mechanism may be neuroprotective in the short-term, continued extracellular release of lysosomal material over the lifetime of an organism may contribute to the propagation and extracellular deposition and spread of pathogenic aggregates in later life as the ability of microglia to degrade this material diminishes. The discovery of Rab27b as one of the most abundantly enriched hits in VPS35 KO H4 cells, both at the transcript and protein level, provides a potential insight into how this process may be upregulated upon Retromer suppression.

Overall, our data emphasises the central importance of Retromer in controlling endolysosomal pathway function beyond its classical role in mediating the sequence-dependent retrieval of integral membrane proteins. This role of Retromer at the nexus of endolysosomal biology likely lies at the heart of its neuroprotective role and dysregulation in a number of neurodegenerative diseases. More generally, our integrated multi-omic approach illustrates a powerful quantitative methodology through which to explore additional avenues for examining the dysregulation of the endo-lysosomal system observed in neurodegenerative disease.

## Methods

### Ethics oversight

The mouse breeding, maintenance, and experimental procedures were all approved by the Institute of Animal Care and Use Committees at the Case Western Reserve University, according to the National Institute of Health (NIH) guidelines.

### Antibodies

Primary antibodies include: β-Actin (Sigma-Aldrich; A1978; clone AC-15; 1:2000 Western blot (WB)), APP (Abcam; Y188; ab32136; 1:200 immunofluorescence (IF)), ATP7A (Santa Cruz; D-9, sc-376467; 1:1000 WB), Cathepsin D (Proteintech; Clone, 21327-1-AP, 1:1000 WB), Cathepsin D (Merck; 219361; 1:200 IF), CI-MPR (Abcam; ab124767; clone EPR6599, 1:1000 WB, 1:400 immunofluorescence (IF)), EEA1 (Cell Signalling; 610456; clone 14; 1:200 IF), EGFR (Cell Signalling Technologies; 2232S; WB 1:1000), EGFR pY1068 (Cell Signalling Technologies; 3777S; WB1:1000), GFP (Roche; 11814460001; clones 7.1/13.1; 1:1000 WB), GLUT1 (Abcam; EPR3915; ab115730; 1:1000 WB; 1:50 IF), HA (Biolegend; 901502; Clone 16B12; 1:1000 WB, 1:200 IF),, LAMP1 (Developmental Studies Hybridoma Bank; AB_2296838; clone H4A3; 1:400 IF) LAMP1 (Abcam; ab21470; 1:200 IF), LAMP1 (Cell Signalling Technologies; CS4H11; 1:1000 WB), p62 (SQSTM1) (BD Transduction Laboratories; 610832; 1:1000 WB, 1:200 IF), Pmp70 (Sigma-Aldrich; 70-18; SAB4200181; 1:1000 WB), Rab7 (Abcam; EPR7589; ab137029; 1:200 IF), Rab27b (Proteintech; 13412-1-AP, 1:500 WB), TGN46 (Bio-Rad; AHP500G; 1:400 IF), TfnR (Santa Cruz; H68.4; sc-65883; 1:1000 WB), VPS29 (Santa Cruz; D-1; sc-398874; 1:500 WB), VPS35 (Abcam; ab157220; clone EPR11501(B); 1:1000 WB).

Secondary antibodies: For Western blotting, 680 nm and 800 nm donkey anti-mouse and anti-rabbit fluorescent secondary antibodies (Invitrogen, A-21057, A3275—1:20,000). For immunofluorescence, 405, 488 nm, 568 nm and 647 nm AlexaFluor-labelled anti-mouse and anti-rabbit secondary antibodies (Invitrogen, A32753, A32731, A10037, A10042, A32849, A32787, A32795—1:400). For mouse samples donkey anti-rat 488 nm Alexa Fluor- or Cy3-conjugated secondary antibodies (Jackson Immunoresearch, 712-156-150, 712-546-150) were used. 0.5 µg/mL 4′, 6-diamidino-2-phenylindole dihydrochloride (DAPI; Sigma-Aldrich, D8417) was added to secondary antibody mixtures to label DNA.

### Animals

All mice were used in the study were in a C57BL/6 genetic background. For both IUE and primary cortical neuron cultures, mothers were 8–12 weeks old. For primary cortical neuronal culture experiments, neurons were isolated at day E18.5. For IUE experiments, embryos were electroporated at E15.5 or E18.5 and neocortical brain sections were taken at P14 or P30. As it is a challenge task to identify sex in mouse embryos, the data were presented without sex-based analysis. The Vps35^f/f^ mice were generated by injecting Vps35^tm1a(EUC^°^MM)Hmgu^ embryonic stem (ES) cells (KO-first promoter-driven reporter-tagged insertion with conditional potential, purchased from International Mouse Phenotyping Consortium (IMPC), Germany) into the inner cell mass of C57BL/6 J blastocysts. In Vps35^tm1a(EUCOMM)Hmgu^ ES cell, the Vps35 gene contains loxP sites flanking exon 6. The injected blastocysts were then implanted into the uterus of pseudo-pregnant foster mothers for further mouse development^96^. The mice were maintained in cages with ad libitum access to water and standard food, which were located in a 12 h light/dark cycle animal room (with room temperature ~22 °C, and humidity at 40–60%) at the animal facility of Case Western Reserve University (CWRU) with standard food and water provided and maintained on a 12 h dark/ light cycle.

### Plasmids

The pCAG-GFP and pCAG-Cre plasmids were purchased from Addgene. The pCAG-Cre-2A-GFP or BFP plasmids were generated by insertion of the P2A domain to the C terminus of Cre, followed by GFP or BFP cDNA, as described previously. The miRNA-Vps35-EmGFP expression vector was first generated by the BLOCK-iT Lentiviral miR RNA expression System (Invitrogen, Carlsbad, CA) according to the manufacturer’s instruction. The target sequence 5′-AGGTGTAAATGTGGAACGTTA-3′, were used for miRNA-Vps35. The miRNA-VPS35-eBFP was then constructed by replacing the EmGFP in the miRNA-Vps35-EmGFP expression vector with eBFP sequence from pBad-eBFP2^20^.

### Primary cortical neuron culture and transfection

Mouse cortical neurons were plated on poly-L-lysine coated coverslip at 50,000–80,000 cells/cm2 in Neurobasal medium (Invitrogen, Carlsbad, CA) containing B-27 (Invitrogen), 2 mM Glutamax-I (Invitrogen), and 2.5% FBS (HyClone, Logan, UT). On the second day in culture (DIV 2), medium was changed to serum starve condition. At DIV3, the neurons were transfected with the indicated plasmids using standard Calcium Phosphate method. At DIV7, the neurons were fixed with 4% PFA, 4% sucrose in PBS for immunostaining analysis^21,97^.

### In utero electroporation (IUE)

The pregnant mice at E15.5 or E18.5 were anesthetised and maintained with 2% isoflurane inhalation and subjected to an abdominal incision to expose the uterus. The plasmid (at a final concentration of ~1 μg/μl), mixed with fast green (0.1 mg/ml, Sigma-Aldrich), were microinjected into the lateral ventricle of the embryos using a glass capillary. The embryos were electroporated with five 50 ms pulses at 45 V with a 950 ms interval through ECM-830 (BTX, Holliston, MA), and then gently reinserted into the pregnant mother’s abdominal cavity, and the abdomen wall and skin were sutured with a surgical needle. These IUE pregnant mice were warmed in an incubator until they regained consciousness and the pups were reared to the indicated postnatal stages. The pups at P14 or P30 derived from the IUE embryos were fixed by cardiac perfusion with 4% paraformaldehyde in 0.1 M phosphate buffer, pH 7.4. Their brains were removed and soaked in the fixative for 2–4 h. After rinsing with phosphate-buffered saline (PBS), their coronal vibratome sections (35–100 μm in thickness) were prepared and used for immunohistochemical staining analyses^21,98^.

### Cell Culture

HeLa and HEK293T cells were sourced from the American Type Culture Collection (ATCC). H4 neuroglioma cells were a gift from Dr Helen Scott and Professor James Uney (University of Bristol). Clonal VPS35 KO HeLa and H4 cell lines were generated with the gRNA sequence 5′-GTGGTGTGCAACATCCCTTG-3′ targeting exon 5 of *VPS35*^18,19^. VPS29 KO cells were generated by transfecting cells with gRNA sequences targeting the sequences 5′-GGACATCAAGTTATTCCAT-3′ and 5′-GGCAAACTGTTGCACCGGTG-3′ within exons 2 and 3 of *VPS29*, respectively.

Cells were grown in Dulbecco’s Modified Eagle Medium (DMEM; Sigma-Aldrich), supplemented with 10% (vol/vol) foetal bovine serum (FBS) (Sigma-Aldrich) and penicillin/streptomycin (Gibco). Cells were transduced with HIV-1-based lentiviruses for stable expression (construct of interest in pXLG3/pLVX/pLJC5^25^ plasmid backbone, and pCMV-dR8.91 packing plasmid) pseudotyped with vesicular stomatitis virus (VSV)-G envelope plasmid (pMDG2). HEK293T cells were transfected with the constituent plasmids using polyethyleneimine (PEI) transfection, then lentiviral particles were harvested after 48 h. H4 cells were seeded into a plate, then transduced with lentivirus following adherence. For pLVX- and pLJC5-expressing cells, 3 μg/mL puromycin dihydrochloride was used for selection.

For amino acid starvation experiments, cells were plated the day before starvation. The culture media (DMEM containing 10% FBS and amino acids) was removed, followed by three PBS washes, and replaced with DMEM lacking amino acids and growth serum for the indicated timepoints. For re-feeding, the starvation media was removed and replaced with DMEM containing amino acids but lacking FBS.

### LysoIP

All equipment was pre chilled and all experimentation was performed at 4 °C. Cells were washed twice in ice-cold PBS and harvested by scraping into 5 ml of KBPS (136 mM KCl, 10 mM KH_2_PO_4_ – pH to 7.5 using KOH) containing freshly added 5 mM TCEP (Thermo #77720). Cells were pelleted by centrifugation at 270*g* for 10 min, re-suspended in 1 ml of lysis buffer KPBS + TCEP and protease/phosphatase inhibitors, prior to mechanical lysis by 6 passages through a 23 G needle. Cell debris was pelleted by centrifugation at 700*g* for 10 min. An aliquot of the lysate was removed to represent the whole cell lysate (treated with Triton TX-100 to a final concentration of 1% and an additional centrifugation at 18,400*g* for 10 min to remove insoluble debris). Lysate volumes were re-adjusted to 1 ml using lysis buffer and added to KPBS washed (x3) magnetic anti-HA beads (Thermo #88837) and gently rotated for 15 min. Beads were pelleted using a magnetic rack and three times washed in KPBS + TCEP for 5 mins with gentle rotation. Beads were pelleted on a magnetic rack and all trace of washing buffer removed, prior to re-suspension in RIPA buffer (10 mM Triz pH7.5, 150 mM NaCl, 1% TX100, 1% Deoxycholate, Protease and Phosphatase inhibitors) and incubation for 15 min with gentle rotation. Beads were pelleted on a magnetic rack and the eluate (solubilised lysosomal material) removed for subsequent analyses.

### Surface biotinylation

All buffers were pre-chilled to 4 °C. Cells were washed twice in ice-cold PBS prior to immersion in ice-cold PBS (pH7.7) containing 200 µg/ml EZ-link sulfo-NHS-SS-biotin (Thermo Scientific #A39258) for 30 min with gentle agitation at 4 °C. To remove excess biotin, cells were washed in 1x PBS followed by 1x in Quench buffer (50 mM Tris, 100 mM NaCl, final pH7.5) prior to a 10 min incubation in quench buffer with gentle agitation. Cells were lysed by scraping in PBS (2% TX100 and protease inhibitor tablets) prior to pelleting of insoluble debris by centrifugation (14k for 10 min). An aliquot of the subsequent cleared lysate was retained to represent the whole cell fraction and the remainder added to pre-washed (in lysis buffer) streptavidin beads (Streptavidin sepharose – Cytiva #17511301). Precipitation of biotinylated cell surface proteins proceeded for 30 min at 4 °C, prior to 1x wash in PBS + 1% TX100, 1x wash in PBS + 1% TX100 and 1 M NaCl and a final wash in PBS. Biotin precipitated beads were pelleted by centrifugation and all traces of wash buffer removed prior to subsequent analyses.

### Quantitative western blotting

Bicinchoninic acid (BCA) assay (Pierce, 23225) or 660 nm assay (Pierce, 22662) was used to determine protein concentration according to the manufacturer’s instructions. NuPAGE 4–12% gradient Bis-Tris precast gels (Life Technologies, NPO336) were used for SDS-PAGE, followed by transfer onto methanol-activated polyvinylidine fluoride (PVDF) membrane (Immobilon-FL membrane, pore size 0.45 μm; Millipore, IPFL00010). Membrane was blocked, then sequentially labelled with primary and secondary antibodies. Fluorescence detected by scanning with a LI-COR Odyssey scanner and Image Studio analysis software (LI-COR Biosciences).

### Immunofluorescence microscopy and analysis

HeLa and H4 cells were seeded onto 13 mm coverslips the day before fixation. DMEM was removed, followed by two washes with PBS, then cells were fixed in 4% paraformaldehyde (PFA) (Pierce, 28906) for 20 min at room temperature. To visualise lysosomal tubules, cells were fixed in 8% PFA in 2X microtubule stabilisation buffer (60 mM PIPES pH 6.8, 10 mM EGTA, 2 mM MgCl_2_) added directly to the cell culture media at a 1:1 volume ratio on a 37 °C heat block, then returned to the tissue culture incubator for 15 min. Cells were permeabilised in 0.1% (w/v) saponin (Sigma-Aldrich, 47036) for 5 min followed by blocking with 1% (w/v) BSA, 0.01% saponin in PBS for 15 min. Coverslips were stained with primary antibodies for 1 h, followed by secondary antibodies for 30 min, then mounted onto glass microscope slides with Fluoromount-G (Invitrogen, 00-4958-02).

Confocal microscope images were taken on a Leica SP5-II confocal laser scanning microscope attached to a Leica DMI 6000 inverted epifluorescence microscope or a Leica SP8 confocal laser scanning microscope attached to a Leica DM l8 inverted epifluorescence microscope (Leica Microsystems), with a 63x UV oil immersion lens, numerical aperture 1.4 (Leica Microsystems, 506192), and acquired using LAS AF software (Leica Microsystems). For the Leica SP8 microscope, ‘lightning’ adaptive image restoration was used to generate deconvolved representative images and acquired using LAS X software (Leica Microsystems). Colocalisation and fluorescence intensity analysis was performed using Volocity 6.3 software (PerkinElmer) with automatic Costes background thresholding^99^. Immunofluorescence images were prepared in Volocity 6.3. Electron microscopy figures were prepared in ImageJ.

Neuronal immunostaining and immunohistochemical staining analyses were carried out as described previously^21,98^. In brief, both the fixed cultured neurons and the P14 vibratome brain sections were blocked with 5% normal serum in PBS with 0.1% Triton X-100 and 2% DMSO, and incubated with primary antibodies for 1 to 2 days. After 1–3 h incubation with donkey anti-rat 488 nm AlexaFluor- or Cy3-conjugated secondary antibodies (Jackson Immunoresearch, 712-156-150, 712-546-150) followed by DAPI staining (0.1 μg/ml, Life Technologies), the stained neurons on the coverslips or brain slices were imaged using a confocal laser scanning microscope (Zeiss LSM800).

### Live cell imaging

Live-cell imaging was performed at 37 °C with cells incubated in starvation media (formulated according to the Gibco recipe for high-glucose DMEM, omitting amino acids/FCS prior to filtration through a 0.22 μm filter) or DMEM supplemented with 10% FCS in a CO2 buffered chamber. Fluorescent cells were imaged live on a Olympus Ixplore—SoRa spinning disk confocal system attached to a Olympus IX83 inverted epifluorescence microscope and a Hamamatsu sCMOS camera. Rapid switching between excitation/emission wavelengths facilitated a capture rate of ~2 frames per second.

### Electron Microscopy

H4 cells were seeded onto 13 mm Thermanox Coverslips (Thermo Scientific) the day before fixation. 10 nm BSA-gold (VWR) was ultracentrifuged at 100,000*g* for 1 h at 4 °C, the supernatant discarded, then the pellet was resuspended in 5 mL of complete DMEM media. The cell culture media was replaced with 10 nm BSA-gold-containing media and cells were incubated at 37 °C for 4 h. Cells were fixed in a 2% paraformaldehyde, 2.5% glutaraldehyde and 0.1 M sodium cacodylate solution for 30 min. Cells were then stained using 1% osmium tetroxide, 1.5% potassium ferrocyanide for 1 h before staining was enhanced by incubation with 1% tannic acid in 0.1 M cacodylate buffer for 45 min. Cells were washed, dehydrated through an ethanol series, and infiltrated with Epoxy propane (CY212 Epoxy resin:propylene oxide) before being infiltrated with full CY212 Epoxy resin and subsequently embedded atop pre-baked Epoxy resin stubs. Epoxy was polymerised at 65 °C overnight before Thermanox coverslips were removed using a heat-block. 70 nm sections were cut using a Diatome diamond knife mounted to an ultramicrotome and sections collected to Pioloform-coated copper slot grids. Ultrathin sections were stained with lead citrate. An FEI Tecnai transmission electron microscope at an operating voltage of 80 kV was used to visualise samples, mounted with a Gatan digital camera.

### Proteomics

#### Experimental design

All proteomic experiments were performed with isobaric tandem mass tagging followed by LC-MS/MS quantitative mass spectrometry. For Lyso IP, 7 independent wild-type cells, 3 independent VPS35 KO Clone 15 and Clone 15 VPS35-GFP rescue and 4 independent VPS35 KO Clone 16 and Clone 16 VPS35-GFP rescue samples were quantified, producing 7 independent repeats of the wild-type vs VPS35 KO vs VPS35-GFP. For surface biotinylation, 6 independent wild-type cells, 3 independent VPS35 KO Clone 15 and Clone 15 VPS35-GFP rescue and 3 independent VPS35 KO Clone 16 and Clone 16 VPS35-GFP rescue samples were quantified, producing 6 independent repeats of the wild-type vs VPS35 KO vs VPS35-GFP. For the whole cell and ‘secretome’ proteomics, 3 independent wild-type samples, 1 VPS35 KO Clone 9 and Clone 9 VPS35-GFP, 1 VPS35 KO Clone 15 and Clone 15 VPS35-GFP and 1 VPS35 KO Clone 16 and Clone 16 VPS35-GFP samples were quantified, producing 3 independent repeats of the wild-type vs VPS35 KO vs VPS35-GFP experimental approach.

Samples for whole cell lysate analysis of H4 cells, cells were grown to confluency in a 10 cm plate, then lysed with 1% TX-100 lysis buffer and quantified with a BCA assay. The concentrations and volumes were normalised to a 200 µL volume of 2 mg/mL protein for each sample. To prepare samples for growth media ‘secretome’ analysis, H4 cells were grown in a 6-well plate in DMEM media without FBS for 16 h. The medium was removed and centrifuged at 300*g* for 10 min at 4 °C, then the supernatant was transferred to a fresh microcentrifuge tube and centrifuged at 2000*g* for a further 10 min 4 °C. The corresponding cells in the 6-well plate were lysed and quantified with a BCA assay to normalise media volumes.

The effects of TMT ratio suppression were minimised by pre-fractionation of the TMT-labelled pool and use of SPS-MS3-based acquisition to minimise ratio suppression due to co-isolation of peptides and, where possible, selecting the labelling set up to minimise any effects of channel bleed-through^100^.

#### TMT Labelling and High pH reversed-phase chromatography

For the cell surface proteome analysis, samples on beads were reduced (10 mM TCEP, 55 °C for 1 h), alkylated (18.75 mM iodoacetamide, room temperature for 30 min.) and then digested from the beads with trypsin (2.5 µg trypsin; 37 °C, overnight). Alternatively, 50ug of each sample (whole cell lysate analysis), Bead eluates (LysoIP analysis), or media samples following concentration to 100ul using Amicon Ultra 3 kDa cut-off centrifugal filters (Merck Millipore Ltd.) (secretome analysis) were reduced, alkylated and digested with trypsin, as described above. Following tryptic digestion, the resulting peptides were labelled with Tandem Mass Tag (TMT) ten plex reagents according to the manufacturer’s protocol (Thermo Fisher Scientific, Loughborough, LE11 5RG, UK) and the labelled samples pooled.

The pooled sample was evaporated to dryness, resuspended in 5% formic acid and then desalted using a SepPak cartridge according to the manufacturer’s instructions (Waters, Milford, Massachusetts, USA). Eluate from the SepPak cartridge was again evaporated to dryness and resuspended in buffer A (20 mM ammonium hydroxide, pH 10) prior to fractionation by high pH reversed-phase chromatography using an Ultimate 3000 liquid chromatography system (Thermo Scientific). In brief, the sample was loaded onto an XBridge BEH C18 Column (130 Å, 3.5 µm, 2.1 mm × 150 mm, Waters, UK) in buffer A and peptides eluted with an increasing gradient of buffer B (20 mM Ammonium Hydroxide in acetonitrile, pH 10) from 0 to 95% over 60 min. The resulting fractions (15 for the whole cell lysate analysis, or 5 for the secretome, LysoIP or cell surface proteome analyses) were evaporated to dryness and resuspended in 1% formic acid prior to analysis by nano-LC MSMS using an Orbitrap Fusion Tribrid mass spectrometer (Thermo Scientific).

#### Nano-LC mass spectrometry

High pH RP fractions were further fractionated using an Ultimate 3000 nano-LC system in line with an Orbitrap Fusion Tribrid mass spectrometer (Thermo Scientific). In brief, peptides in 1% (vol/vol) formic acid were injected onto an Acclaim PepMap C18 nano-trap column (Thermo Scientific). After washing with 0.5% (vol/vol) acetonitrile 0.1% (vol/vol) formic acid peptides were resolved on a 250 mm × 75 μm Acclaim PepMap C18 reverse phase analytical column (Thermo Scientific) over a 150 min organic gradient, using 7 gradient segments (1–6% solvent B over 1 min, 6–15% B over 58 min, 15–32%B over 58 min, 32–40%B over 5 min, 40–90%B over 1 min, held at 90%B for 6 min and then reduced to 1%B over 1 min) with a flow rate of 300 nl min^−1^. Solvent A was 0.1% formic acid and Solvent B was aqueous 80% acetonitrile in 0.1% formic acid. Peptides were ionised by nano-electrospray ionisation at 2.0 kV using a stainless-steel emitter with an internal diameter of 30 μm (Thermo Scientific) and a capillary temperature of 275 °C.

All spectra were acquired using an Orbitrap Fusion Tribrid mass spectrometer controlled by Xcalibur 2.1 software (Thermo Scientific) and operated in data-dependent acquisition mode using an SPS-MS3 workflow. FTMS1 spectra were collected at a resolution of 120,000, with an automatic gain control (AGC) target of 200,000 and a max injection time of 50 ms. Precursors were filtered with an intensity threshold of 5000, according to charge state (to include charge states 2–7) and with monoisotopic peak determination set to peptide. Previously interrogated precursors were excluded using a dynamic window (60 s ±10ppm). The MS2 precursors were isolated with a quadrupole isolation window of 1.2 *m/z*. ITMS2 spectra were collected with an AGC target of 10 000, max injection time of 70 ms and CID collision energy of 35%.

For FTMS3 analysis, the Orbitrap was operated at 50 000 resolution with an AGC target of 50 000 and a max injection time of 105 ms. Precursors were fragmented by high energy collision dissociation (HCD) at a normalised collision energy of 60% to ensure maximal TMT reporter ion yield. Synchronous Precursor Selection (SPS) was enabled to include up to 10 MS2 fragment ions in the FTMS3 scan.

#### Data analysis

The raw data files were processed and quantified using Proteome Discoverer software v2.1 (Thermo Scientific) and searched against the UniProt Human database (downloaded January 2022; 178486 sequences) using the SEQUEST HT algorithm. Peptide precursor mass tolerance was set at 10ppm, and MS/MS tolerance was set at 0.6 Da. Search criteria included oxidation of methionine (+15.995 Da), acetylation of the protein N-terminus (+42.011 Da) and Methionine loss plus acetylation of the protein N-terminus (−89.03 Da) as variable modifications and carbamidomethylation of cysteine (+57.021 Da) and the addition of the TMT mass tag (+229.163 Da) to peptide N-termini and lysine as fixed modifications. Searches were performed with full tryptic digestion and a maximum of 2 missed cleavages were allowed. The reverse database search option was enabled and all data was filtered to satisfy false discovery rate (FDR) of 5%.

### RNA-Seq

6 independent wild-type samples, 2 VPS35 KO Clone 9 and Clone 9 VPS35-GFP, 2 VPS35 KO Clone 15 and Clone 15 VPS35-GFP and 2 VPS35 KO Clone 16 and Clone 16 VPS35-GFP samples were quantified, producing 6 independent repeats of the wild-type vs VPS35 KO vs VPS35-GFP experimental approach. H4 cells were grown to confluence in a 6-well plate. Media was removed and cells were washed twice with ice cold PBS. Cells were lysed and RNA was purified using the RNeasy kit (Qiagen) according to the manufacturer’s instructions. RNA concentration was measured using a NanoDrop 1000 machine (Thermo Fisher). Concentrations of all samples were normalised to 50 ng/µL.

Total RNA was quantified using the Qubit 2.0 fluorimetric Assay (Thermo Fisher Scientific). Libraries were prepared from 125 ng of total RNA using the NEGEDIA Digital mRNAseq research grade sequencing service (Next Generation Diagnostics srl)^101^ which included library preparation, quality assessment and sequencing on a NovaSeq 6000 sequencing system using a single-end, 100 cycle strategy (Illumina Inc.).

The raw data were analysed by Next Generation Diagnostics srl proprietary 3’DGE mRNA-seq pipeline (v1.0) which involves a cleaning step by quality filtering and trimming, alignment to the reference genome and counting by gene (https://sourceforge.net/projects/bbmap/)^102,103^. We filtered out all genes having <1 cpm in less than n_min samples. Differential expression analysis was performed using edgeR^104^.

Gene set enrichment analysis (GSEA) of RNA-Seq data was performed using the GSEA software (UC San Diego and Broad Institute) and MSigDB v7.2 database of gene sets. Specifically, the cellular compartment gene ontology gene sets (c5.go.cc.v7.2.symbols.gmt) and Kyoto Encyclopaedia of Genes and Genomes (KEGG) pathway gene sets (c2.cp.kegg.v7.2.symbols.gmt) were used for analysis^105,106^. Gene set networks from GSEA were visualised using Cytoscape 3.3 software with the Enrichment Map plug-in^107^.

### Statistics and bioinformatic analysis

Raw files from mass spectrometry were quantified using Proteome Discoverer software v2.1 (Thermo Fisher). Peptides were searched against the UniProt human proteome database using the SEQUEST algorithm. For normalisation of mass spectrometry data, protein abundances were normalised based on total peptide amount for each sample in Proteome Discoverer 2.1, which sums the peptide group abundances for each sample and determines the maximum sum for all files. A normalisation factor is calculated as the sum of the sample and the maximum sum in all files, and is applied to each sample to normalise the data. For the whole cell proteome, LysoIP and surface biotinylation, the normalised values were used for analysis (Supplementary Data 2). For the growth media “secretome”, the raw values were used (Supplementary Data 1). Where proteins were identified and quantified by an identical group of peptides as the master protein of their protein group, these are designated ‘candidate master proteins’. We then used the annotation metrics for candidate master proteins retrieved from Uniprot to select the best annotated protein which was then designated as master protein. This enables us to infer biological trends more effectively in the dataset without any loss in the quality of identification or quantification. The mass spectrometry data were searched against the human Uniprot database retrieved on 2021-01-14, and updated with additional annotation information on 2021-11-15. To assemble the integrated datasets presented in Supplementary Data 1 and 2, proteins were compared between datasets first using master protein accessions, and secondly using candidate master proteins, to ensure the best possible comparison. RNA data was integrated into the proteomics data using the biomaRt package in R^108^.

For statistical analysis of differential protein abundance between conditions, standard two-tailed paired t-tests were used and false discovery rate (FDR) was calculated using the Benjamini-Hochberg correction. Both statistical metrics are displayed in Supplementary Data 1 and 2. Volcano plots were plotted either the VolcaNoseR webapp^109^. Typically, thresholds of log_2_ fold change of ±0.26 (corresponding to a 1.2-fold enrichment or depletion), and a -log_10_ p-value of 1.3 (corresponding to 0.05) were set, although these thresholds were adjusted based on assessment of data distributions for various experiments. Scatter plots were constructed in GraphPad Prism 9 (LaJolla, CA) software.

Gene ontology analysis was performed using Metascape 3.5^110^ to represent pathway enrichment, DisGeneNET category enrichment, and protein-protein interaction (PPI) networks, using a hypergeometric test and Benjamini-Hochberg correction, and the PANTHER v16.0 classification system^111^ was used to represent cellular component enrichment, using a Fisher’s exact test. Raw gene ontology output data is provided in Supplementary Data 3. The dotted line overlaid on pathway enrichment graph represents a *p* = 0.05 statistical cut-off. PPI networks were visualised using Cytoscape 3.3 software with the Enrichment Map plug-in^107^.

All statistical analysis was performed on data from a minimum of 3 independent experimental repeats. Graphs were prepared in GraphPad Prism 9 or VolcanoseR. Individual datapoints represent independent experimental repeats. For comparisons made against fixed normalised control values, one-sample two-tailed *t* tests were used against a hypothetical mean value of 100, followed by Holm–ídák Post-Hoc Correction. Both *t* test *p*-values and adjusted *p*-values are demonstrated in the source data, and all graphs represent adjusted *p*-values. Graphs are plotted representing the mean value ± the standard error of the mean (SEM) for each experimental condition. *n* represents the number of independent experimental repeats. In all graphs, *p < 0.05, **p < 0.01, ***p < 0.001, ****p < 0.0001.

### Reporting summary

Further information on research design is available in the Nature Portfolio Reporting Summary linked to this article.

## Supplementary information

Supplementary Information

Description of Additional Supplementary Files

Supplementary Data 1

Supplementary Data 2

Supplementary Data 3

Supplementary Data 4

Supplementary Data 5

Supplementary Data 6

Supplementary Data 7

Supplementary Data 8

Supplementary Movie 1

Supplementary Movie 2

Supplementary Movie 3

Supplementary Movie 4

Supplementary Movie 5

Supplementary Movie 6

Supplementary Movie 7

Supplementary Movie 8

Supplementary Movie 9

Supplementary Movie 10

Supplementary Movie 11

Supplementary Movie 12

Reporting Summary

## Data Availability

The datasets generated during this study and minimum datasets required to interpret the data are included in the published article as Supplementary Data 1–8, and in public repositories. The mass spectrometry proteomics data generated in this study have been deposited in the ProteomeXchange Consortium via the PRIDE^112^ partner repository under accession code PXD041323 The RNA-Seq data generated in this study have been deposited in the NCBI Gene Expression Omnibus^113^ under accession code GSE223292 Mass spectrometry data were searched against the human Uniprot database retrieved on 2021-01-14, and updated with additional annotation information on 2021-11-15. Unprocessed blots and statistical data are included as Source Data. Source data are provided with this paper.

## Source data

Source Data
