# Supplementary material for: Multi-omic approach characterises the neuroprotective role of retromer in regulating lysosomal health
Source: Nat Commun. 2023 May 29;14:3086. doi: 10.1038/s41467-023-38719-8 (PMC10227043; doi:10.1038/s41467-023-38719-8)
Supplement: Supplementary file 1 — Supplementary Information [file 41467_2023_38719_MOESM1_ESM.pdf]

SUPPLEMENTARY INFORMATION

Multiomic Approach Characterises the Neuroprotective Role of Retromer in Regulating Lysosomal Health.

James L. Daly[1,11,12,‡], Chris M. Danson[1,11], Philip A. Lewis[2], Lu Zhao[3], Sara Riccardo[4,5], Lucio Di Filippo[4,5], Davide Cacchiarelli[4,6,7], Daehoon Lee[3] Stephen J. Cross[8], Kate J. Heesom[3], Wen-Cheng Xiong[3], Andrea Ballabio[4,7,9], James R. Edgar[10], Peter J. Cullen[1,‡].

[1]School of Biochemistry, Biomedical Sciences Building, University Walk, University of Bristol, BS8 1TD, U.K.

[2]Bristol Proteomics Facility, School of Biochemistry, Biomedical Sciences Building, University Walk, University of Bristol, BS8 1TD, U.K.

[3]Department of Neurosciences, Case Western Reserve University, Cleveland, Ohio, U.S.A.

[4]Telethon Institute of Genetics and Medicine, Armenise/Harvard Laboratory of Integrative Genomics, Pozzuoli, Italy.

[5]Next Generation Diagnostic srl, Pozzuoli, Italy.

[6]Department of Translational Medicine, University of Naples “Federico II”, Naples, Italy.

[7]School for Advanced Studies, University of Naples "Federico II", Naples, Italy.

[8]Wolfson Bioimaging Facility, Faculty of Biomedical Sciences, University of Bristol, Bristol, U.K.

[9]Department of Molecular and Human Genetics and Neurological Research Institute, Baylor College of Medicine, Houston, TX, U.S.A.

[10]Department of Pathology, Cambridge University, Tennis Court Road, Cambridge, U.K.

[11]Contributed equally to this study
[12]Present Address: Department of Infectious Diseases, School of Immunology and Microbial Sciences, Guy’s Hospital, King’s College London, SE1 9RT, U.K.

‡Joint corresponding authors. Correspondence: james.l.daly@kcl.ac.uk, pete.cullen@bristol.ac.uk

Contents: Supplementary Figures and Legends

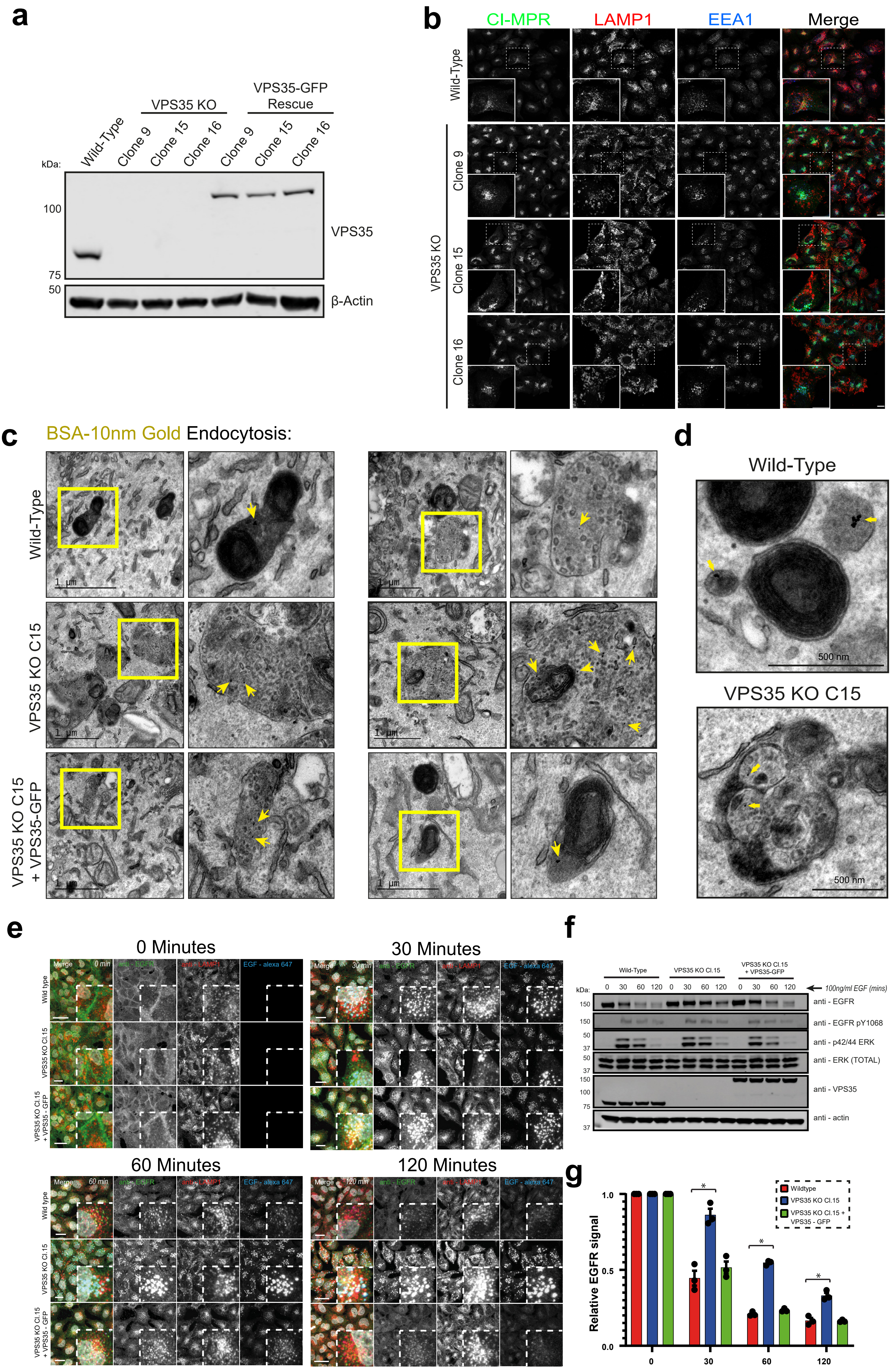
a
VPS35 KO
VPS35-GFP Rescue
Wild-Type
Clone 9
Clone 15
Clone 16
Clone 9
Clone 15
Clone 16
kDa:
100
75
50
VPS35
β-Actin
b
CI-MPR
LAMP1
EEA1
Merge
Wild-Type
VPS35 KO
Clone 9
Clone 15
Clone 16
c
BSA-10nm Gold Endocytosis:
Wild-Type
VPS35 KO C15
VPS35 KO C15 + VPS35-GFP
1 µm
d
Wild-Type
VPS35 KO C15
500 nm
e
0 Minutes
30 Minutes
60 Minutes
120 Minutes
Merge
anti - EGFR
anti - LAMP1
EGF - alexa 647
Wild type
VPS35 KO Cl.15
VPS35 KO Cl.15 + VPS35 - GFP
f
Wild-Type
VPS35 KO Cl.15
VPS35 KO Cl.15 + VPS35-GFP
kDa:
0 30 60 120
100ng/ml EGF (mins)
anti - EGFR
anti - EGFR pY1068
anti - p42/44 ERK
anti - ERK (TOTAL)
anti - VPS35
anti - actin
150
50
37
100
75
g
Relative EGFR signal
1.0
0.5
0.0
0
30
60
120
Wildtype
VPS35 KO Cl.15
VPS35 KO Cl.15 + VPS35 - GFP
*

Supplementary Figure 1. Validation of VPS35 KO Clones and Lysosomal Morphology Defects

(A) Western blot validation of VPS35-GFP expression in VPS35 KO H4 cells. The presence of the VPS35-GFP construct is indicated by increased molecular weight of VPS35, corresponding to GFP. Data representative of 3 independent repeats. kDa = kilodaltons. **(B)** Immunofluorescence staining of three independent VPS35 KO clones with altered CI-MPR, LAMP1 and EEA1 compartment morphology. Scale bar = 20 µm, zoom scale bar = 5 µm. Data representative of 3 independent repeats. **(C-D)** Wild-type, VPS35 KO and VPS35-GFP-expressing rescue cells were incubated for 4 hours with 10nm BSA-gold prior to fixation and processing for electron microscopy. Transmission electron micrographs of internalized gold particles. Scale bars: 1 µm (C) and 500 nm (D). Data representative of one repeat. **(E)** EGFR is sorted to lysosomes but inefficiently degraded in VPS35 KO. Cells were serum starved to distribute EGFR at the plasma membrane prior to receptor activation via addition of EGF-Alexa-Fluor 647 (100 ng/ml) for the denoted time points and subsequent fixation and immuno-staining for EGFR and LAMP1. Scale bars: 20 µm. Data representative of 3 independent repeats. **(F)** The kinetics of EGFR degradation are perturbed in VPS35 KO relative to control and rescue cell lines. Cells were starved as above prior to stimulation with EGF (100 ng/ml) for the indicated time periods and immuno-blotting with anti-EGFR, EGFR pY1068, total ERK, phospho ERK (p42/44), VPS35 and β-actin. Scale bars = 20 µm. **(G)** Quantification of EGFR degradation time course over n=3 independent experiments. Means +/- SEM, two-way ANOVA with Tukey's multiple comparisons tests. At 30 and 60 min EGF stimulation: wild-type vs VPS35 KO p = <0.0001 and VPS35-GFP vs VPS35 KO p = <0.0001, at 120 min: wild-type vs VPS35 KO p = 0.0037 and VPS35-GFP vs VPS35 KO p = 0.0002.

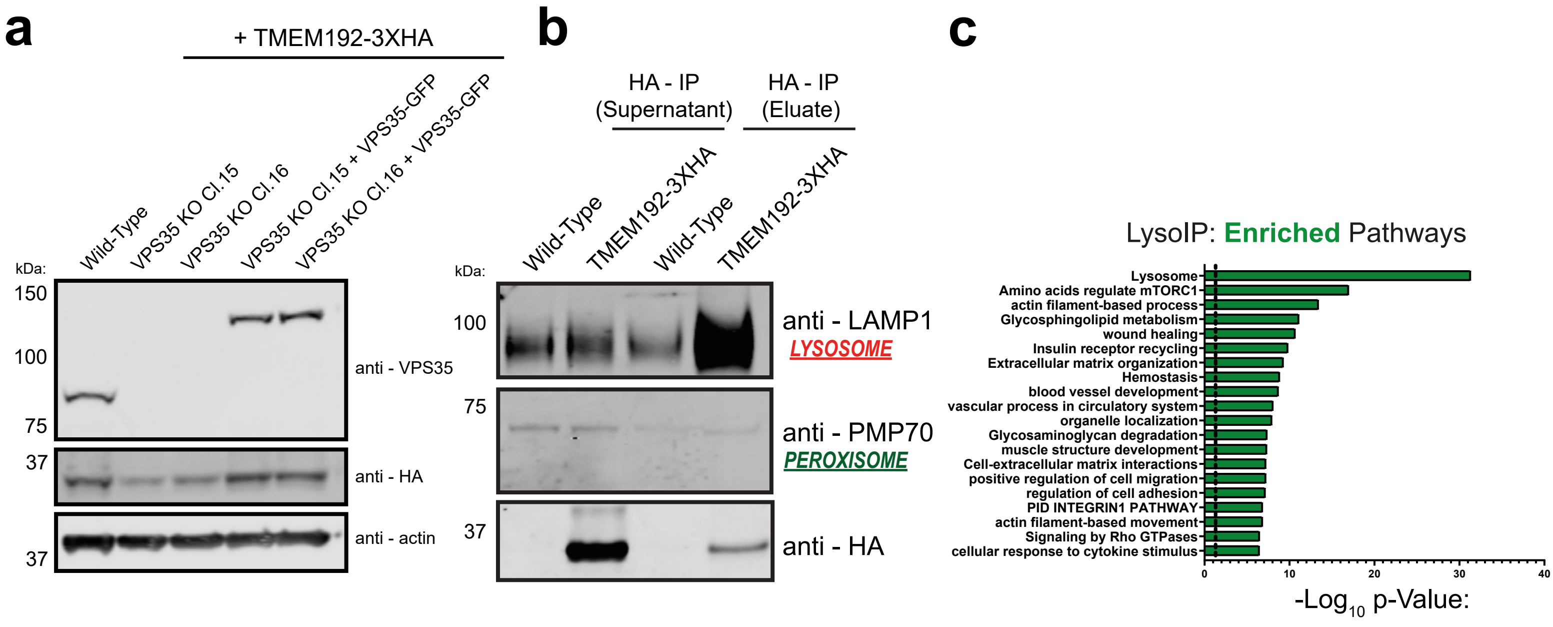
a
+ TMEM192-3XHA
Wild-Type
VPS35 KO Cl.15
VPS35 KO Cl.16
VPS35 KO Cl.15 + VPS35-GFP
VPS35 KO Cl.16 + VPS35-GFP
kDa:
150
100
75
37
37
anti - VPS35
anti - HA
anti - actin
b
HA - IP
(Supernatant)
HA - IP
(Eluate)
Wild-Type
TMEM192-3XHA
Wild-Type
TMEM192-3XHA
kDa:
100
75
37
anti - LAMP1
LYSOSOME
anti - PMP70
PEROXISOME
anti - HA
c
LysoIP: Enriched Pathways
Lysosome
Amino acids regulate mTORC1
actin filament-based process
Glycosphingolipid metabolism
wound healing
Insulin receptor recycling
Extracellular matrix organization
Hemostasis
blood vessel development
vascular process in circulatory system
organelle localization
Glycosaminoglycan degradation
muscle structure development
Cell-extracellular matrix interactions
positive regulation of cell migration
regulation of cell adhesion
PID INTEGRIN1 PATHWAY
actin filament-based movement
Signaling by Rho GTPases
cellular response to cytokine stimulus
0
10
20
30
40
-Log10 p-Value:

Supplementary Figure 2. Development of LysoIP methodology.

(A) Representative western blot depicting TMEM192-3xHA expression in cell lines. Data representative of 3 independent repeats. kDa = kilodaltons. **(B)** LysoIP specifically enriches for lysosomal markers. Western blot probing for lysosomal and peroxisomal compartment markers in LysoIP samples derived from wild-type and TMEM192-3xHA expressing H4. Data representative of 3 independent repeats. **(C)** Pathway analysis of significantly enriched pathways in TMEM192-3xHA-expressing cells subjected to LysoIP relative to wild-type control H4 cells, hypergeometric test.

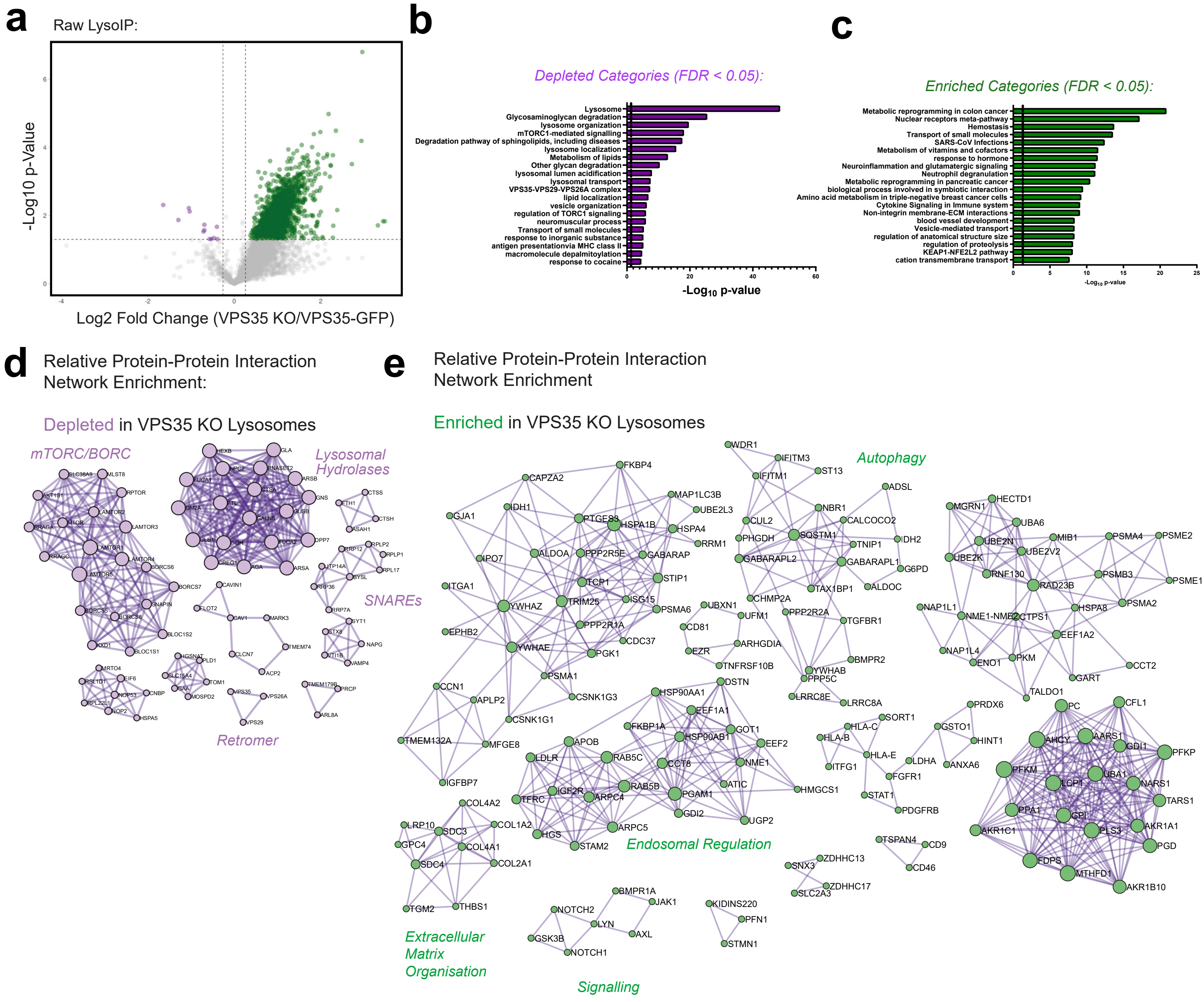
a
Raw LysoIP:
-Log10 p-Value
Log2 Fold Change (VPS35 KO/VPS35-GFP)
b
Depleted Categories (FDR < 0.05):
Lysosome
Glycosaminoglycan degradation
lysosome organization
mTORC1-mediated signalling
Degradation pathway of sphingolipids, including diseases
lysosome localization
Metabolism of lipids
Other glycan degradation
lysosomal lumen acidification
lysosomal transport
VPS35-VPS29-VPS26A complex
lipid localization
vesicle organization
regulation of TORC1 signaling
neuromuscular process
Transport of small molecules
response to inorganic substance
antigen presentationvia MHC class II
macromolecule depalmitoylation
response to cocaine
-Log10 p-value
c
Enriched Categories (FDR < 0.05):
Metabolic reprogramming in colon cancer
Nuclear receptors meta-pathway
Hemostasis
Transport of small molecules
SARS-CoV Infections
Metabolism of vitamins and cofactors
response to hormone
Neuroinflammation and glutamatergic signaling
Neutrophil degranulation
Metabolic reprogramming in pancreatic cancer
biological process involved in symbiotic interaction
Amino acid metabolism in triple-negative breast cancer cells
Cytokine Signaling in Immune system
Non-integrin membrane-ECM interactions
blood vessel development
Vesicle-mediated transport
regulation of anatomical structure size
regulation of proteolysis
KEAP1-NFE2L2 pathway
cation transmembrane transport
-Log10 p-value
d
Relative Protein-Protein Interaction Network Enrichment:
Depleted in VPS35 KO Lysosomes
mTORC/BORC
Lysosomal Hydrolases
SNAREs
Retromer
e
Relative Protein-Protein Interaction Network Enrichment
Enriched in VPS35 KO Lysosomes
Autophagy
Endosomal Regulation
Extracellular Matrix Organisation
Signalling

Supplementary Figure 3. Additional LysoIP Proteomic Analysis.

(A) Bulk lysosomal content is enriched in VPS35 KO cells. Volcano plot of raw VPS35 KO/VPS35-GFP protein abundances. 1024 and 6 proteins were significantly enriched and depleted, respectively, in VPS35 KO LysoIP compared to both wild-type and rescue samples (Log2 fold change ± 0.26, $p < 0.05$), two-tailed paired t-tests. Magenta – proteins significantly depleted in VPS35 KOs, green – proteins significantly enriched in VPS35 KOs. **(B-C)** Gene ontology analysis of depleted (E) and enriched (F) proteins on the lysosomal proteome passing a 5% FDR cutoff calculated by Benjamini-Hochberg correction, hypergeometric test. **(D-E)** Protein-protein interaction networks of significantly depleted (B) and enriched (C) proteins in the normalised VPS35 KO LysoIP relative to wild-type and VPS35-GFP samples. Magenta – proteins significantly depleted in VPS35 KOs, green – proteins significantly enriched in VPS35 KOs.

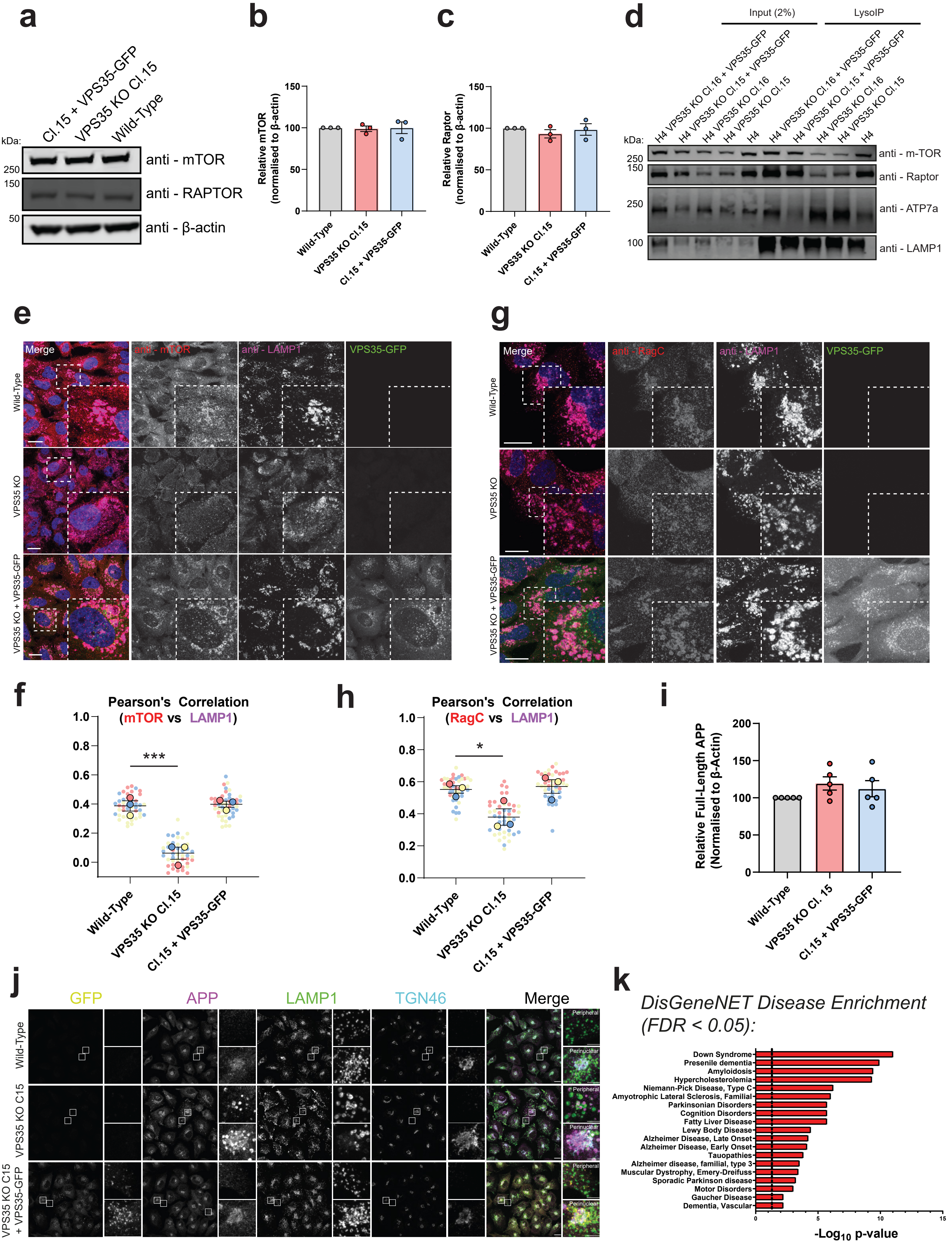
a
Cl.15 + VPS35-GFP
VPS35 KO Cl.15
Wild-Type
kDa:
250
150
50
anti - mTOR
anti - RAPTOR
anti - β-actin
b
Relative mTOR
(normalised to β-actin)
150
100
50
0
Wild-Type
VPS35 KO Cl.15
Cl.15 + VPS35-GFP
c
Relative Raptor
(normalised to β-actin)
150
100
50
0
Wild-Type
VPS35 KO Cl.15
Cl.15 + VPS35-GFP
d
Input (2%)
LysoIP
H4 VPS35 KO Cl.16 + VPS35-GFP
H4 VPS35 KO Cl.15 + VPS35-GFP
H4 VPS35 KO Cl.16
H4 VPS35 KO Cl.15
H4
H4 VPS35 KO Cl.16 + VPS35-GFP
H4 VPS35 KO Cl.15 + VPS35-GFP
H4 VPS35 KO Cl.16
H4 VPS35 KO Cl.15
H4
kDa:
250
150
250
100
anti - m-TOR
anti - Raptor
anti - ATP7a
anti - LAMP1
e
Merge
anti - mTOR
anti - LAMP1
VPS35-GFP
Wild-Type
VPS35 KO
VPS35 KO + VPS35-GFP
g
Merge
anti - RagC
anti - LAMP1
VPS35-GFP
Wild-Type
VPS35 KO
VPS35 KO + VPS35-GFP
f
Pearson's Correlation
(mTOR vs LAMP1)
1.0
0.8
0.6
0.4
0.2
0.0
***
Wild-Type
VPS35 KO Cl.15
Cl.15 + VPS35-GFP
h
Pearson's Correlation
(RagC vs LAMP1)
1.0
0.8
0.6
0.4
0.2
0.0
*
Wild-Type
VPS35 KO Cl.15
Cl.15 + VPS35-GFP
i
Relative Full-Length APP
(Normalised to β-Actin)
200
150
100
50
0
Wild-Type
VPS35 KO Cl. 15
Cl.15 + VPS35-GFP
j
GFP
APP
LAMP1
TGN46
Merge
Wild-Type
VPS35 KO C15
VPS35 KO C15
+ VPS35-GFP
Peripheral
Perinuclear
k
DisGeneNET Disease Enrichment
(FDR < 0.05):
Down Syndrome
Presenile dementia
Amyloidosis
Hypercholesterolemia
Niemann-Pick Disease, Type C
Amyotrophic Lateral Sclerosis, Familial
Parkinsonian Disorders
Cognition Disorders
Fatty Liver Disease
Lewy Body Disease
Alzheimer Disease, Late Onset
Alzheimer Disease, Early Onset
Tauopathies
Alzheimer disease, familial, type 3
Muscular Dystrophy, Emery-Dreifuss
Sporadic Parkinson disease
Motor Disorders
Gaucher Disease
Dementia, Vascular
0
5
10
15
-Log10 p-value

Supplementary Figure 4. VPS35 KO alters the recruitment of mTORC1 and APP to lysosomes.

(A). Cell lysates from indicated cell lines were immuno-blotted using anti–mTOR and -Raptor antibodies. kDa = kilodaltons. **(B, C)** Quantification of *n* = 3 independent experiments, means+/- SEM, one-sample two-tailed t-test with Holm-Šídák correction, corrected p-values: wild-type vs VPS35 KO Cl. 15 0.7691, WT vs Cl.15 VPS35-GFP 0.9982. Equal samples were loaded onto two gels and processed in parallel. Anti-mTOR and anti-β-actin were immunoblotted on the same gel, with anti-Raptor on another gel and normalised to β-actin levels. **(D)** Wild-type, VPS35 KO Clones 15 and 16 and corresponding VPS35-GFP rescues expressing TMEM192-3xHA were subjected to LysoIP followed by Western blotting. **(E-H)** Cells were amino acid starved (2h), prior to re-feeding in D-MEM (15 min) and subsequent fixation and immuno-staining with indicated antibodies. mTOR and RagC colocalisation was quantified by measuring Pearson's correlation co-efficient between the respective fluorescent signals. n=3 independent experiments, means +/- SEM, one way ANOVA with Dunnett's multiple comparisons tests. (mTOR) Wild-type vs VPS35 KO Cl.15, p= 0.0009 and Wild-type vs VPS35 KO Cl.15 + VPS35-GFP, p= 0.9573. (RagC) Wild-type vs VPS35 KO Cl.15, p= 0.0424 and Wild-type vs VPS35 KO Cl.15 + VPS35-GFP, p = 0.9274. Scale bars: 20 μm. Datapoints coloured by independent repeat. **(I)** Quantification of full-length APP levels in wild-type, VPS35 KO and VPS35-GFP-expressing rescue cells, displayed in Figure 4K. n=5 independent experiments, means+/- SEM, one-sample two-tailed t-tests with Holm-Šídák correction, adjusted p = 0.1801 (VPS35 KO Cl.15), 0.3186 (Cl.15 + VPS35-GFP). **(J)** Immunofluorescence staining of wild-type, VPS35 KO Clone 15 and Clone 15 VPS35-GFP rescue cells stained with anti-APP -LAMP1 and -TGN46 antibodies. Insets depict examples of perinuclear APP (centred on the *trans*-Golgi network (TGN)) and peripheral APP. Scale bar = 20 μm, zoom scale = 5 μm**.** Data representative of 3 independent repeats.**(K)** Enrichment of selected DisGeneNET disease categories represented by < 5%FDR enriched proteins in the VPS35 KO LysoIP dataset, calculated by Benjamini-Hochberg correction (log_2 fold change ± 0.26, FDR <0.05), relative to both wild-type and VPS35-GFP-expressing control conditions, hypergeometric test.

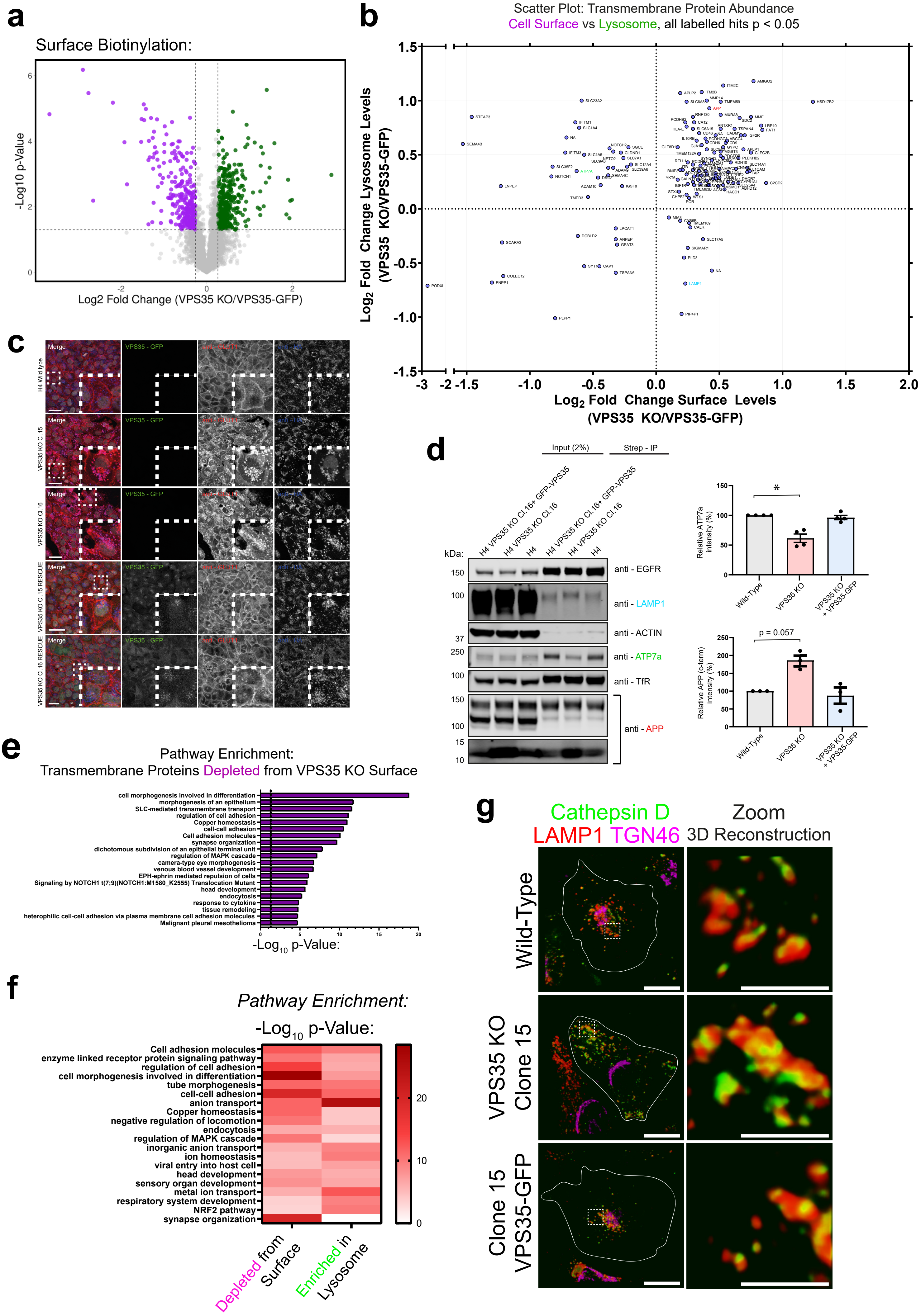
a
Surface Biotinylation:
-Log10 p-Value
Log2 Fold Change (VPS35 KO/VPS35-GFP)
b
Scatter Plot: Transmembrane Protein Abundance
Cell Surface vs Lysosome, all labelled hits p < 0.05
Log2 Fold Change Lysosome Levels (VPS35 KO/VPS35-GFP)
Log2 Fold Change Surface Levels (VPS35 KO/VPS35-GFP)
c
Merge
VPS35 - GFP
H4 Wild type
VPS35 KO Cl.15
VPS35 KO Cl.16
VPS35 KO Cl.15 RESCUE
VPS35 KO Cl.16 RESCUE
d
Input (2%)
Strep - IP
H4 VPS35 KO Cl.16+ GFP-VPS35
H4 VPS35 KO Cl.16
H4
kDa:
anti - EGFR
anti - LAMP1
anti - ACTIN
anti - ATP7a
anti - TfR
anti - APP
Relative ATP7a intensity (%)
Relative APP (c-term) intensity (%)
p = 0.057
Wild-Type
VPS35 KO
VPS35 KO + VPS35-GFP
e
Pathway Enrichment:
Transmembrane Proteins Depleted from VPS35 KO Surface
cell morphogenesis involved in differentiation
morphogenesis of an epithelium
SLC-mediated transmembrane transport
regulation of cell adhesion
Copper homeostasis
cell-cell adhesion
Cell adhesion molecules
synapse organization
dichotomous subdivision of an epithelial terminal unit
regulation of MAPK cascade
camera-type eye morphogenesis
venous blood vessel development
EPH-ephrin mediated repulsion of cells
Signaling by NOTCH1 t(7;9)(NOTCH1:M1580_K2555) Translocation Mutant
head development
endocytosis
response to cytokine
tissue remodeling
heterophilic cell-cell adhesion via plasma membrane cell adhesion molecules
Malignant pleural mesothelioma
-Log10 p-Value:
f
Pathway Enrichment:
-Log10 p-Value:
Cell adhesion molecules
enzyme linked receptor protein signaling pathway
regulation of cell adhesion
cell morphogenesis involved in differentiation
tube morphogenesis
cell-cell adhesion
anion transport
Copper homeostasis
negative regulation of locomotion
endocytosis
regulation of MAPK cascade
inorganic anion transport
ion homeostasis
viral entry into host cell
head development
sensory organ development
metal ion transport
respiratory system development
NRF2 pathway
synapse organization
Depleted from Surface
Enriched in Lysosome
g
Cathepsin D
LAMP1 TGN46
Zoom
3D Reconstruction
Wild-Type
VPS35 KO Clone 15
Clone 15 VPS35-GFP

Supplementary Figure 5. Proteomic Profiling of the VPS35 KO Cell Surface and Lysosomal Proteomes

(A) Volcano plot of VPS35 KO/VPS35-GFP protein abundances in the cell surface proteome. 270 and 199 proteins were significantly enriched and depleted, respectively (Log2 fold change ± 0.26, $p < 0.05$), two-tailed paired t-tests. Magenta – proteins significantly depleted in VPS35 KOs, green – proteins significantly enriched in VPS35 KOs. **(B)** Scatter plot of VPS35 KO/VPS35-GFP protein abundances in the cell surface proteome (x-axis) versus the LysoIP proteome (y-axis). Only significantly proteins in both experiments are displayed and labelled (Log_2 fold change ± 0.26, $p < 0.05$), two-tailed paired t-tests. Coloured labels indicate proteins immunoblotted in (D). **(C)** Imaging of endogenous GLUT1 steady-state distribution establishing that re-expression of VPS35-GFP rescues the missorting to lysosomes observed in VPS35 KO H4 cells. Data representative of 3 independent repeats. **(D)** Cell surface abundance of APP-CTF is elevated in VPS35 KO H4 cells. Cell surface precipitates from indicated cell lines were immuno-blotted using anti – APP (full length and CTF), EGFR, TfR and LAMP1 (confirmation of enrichment of surface proteome), β-actin and ATP7a. Quantification of APP-CTF (n=3) and ATP7a (n=4) cell surface abundances. Means +/- SEM, one-sample two-tailed t-tests with Holm-Šídák correction, ATP7A adjusted p = 0.0246 (VPS35 KO Cl.16), 0.4034 (VPS35 KO Cl.16 + VPS35-GFP), APP adjusted p = 0.0568 (VPS35 KO Cl.16), 0.6356 (VPS35 KO Cl.16 + VPS35-GFP). kDa = kilodaltons. **(E)** Pathway analysis of significantly depleted pathways in the VPS35 KO transmembrane cell surface proteome relative to wild-type and VPS35-GFP rescue controls, hypergeometric test. **(F)** Pathway enrichment analysis of significantly depleted pathways represented by transmembrane proteins significantly depleted from the VPS35 KO cell surface proteome, and transmembrane proteins significantly enriched in the VPS35 KO LysoIP proteome relative to wild-type and VPS35-GFP rescues, hypergeometric test. **(G)** Cathepsin D localises to LAMP1-positive compartments in VPS35 KO H4 cells. Immunofluorescence microscopy of wild-type, VPS35 KO and VPS35-GFP rescue H4 cells stained with anti-CTSD, -LAMP1 and -TGN46 antibodies. Scale bar = 20 μm, zoom 3D reconstruction scale bar = 5 μm. Data representative of 3 independent repeats.

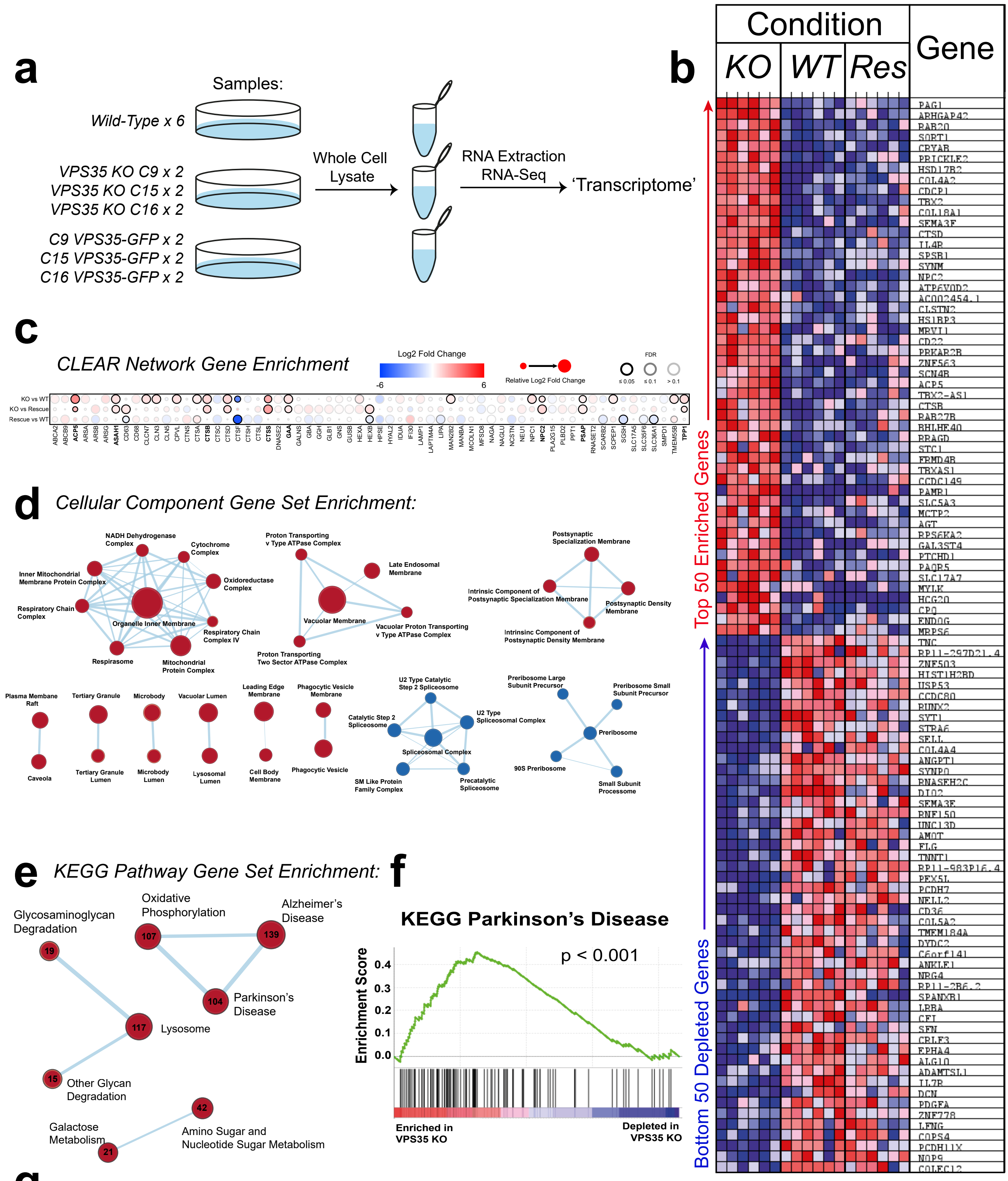

g

VPS35 KO Enrichment Rank	Gene Set	Size	Net Enrichment Score	Nominal p-Value	FDR q-Value
1	KEGG_OXIDATIVE_PHOSPHORYLATION	107	2.39	<0.001	<0.001
2	KEGG_LYSOSOME	117	2.38	<0.001	<0.001
7	KEGG_PARKINSONS_DISEASE	104	1.96	<0.001	0.005
19	KEGG_ALZHEIMERS_DISEASE	139	1.66	0.002	0.057
37	KEGG_AUTOIMMUNE_THYROID_DISEASE	25	1.36	0.093	0.228
38	KEGG_HUNTINGTONS_DISEASE	158	1.35	0.023	0.226

Supplementary Figure 6. Transcriptional Profiling of VPS35 KO Cells

(A) Schematic of the RNA-seq experimental design. 6 wild-type samples, 6 VPS35 KO samples (2 each of VPS35 KO clones 9, 15 and 16) and 6 VPS35-GFP rescue samples (2 each of VPS35-GFP rescue clones 9, 15 and 16) were analysed. **(B)** Heatmap depicting the top 50 up- and down-regulated genes in VPS35 KO samples relative to both wild-type and rescue cells. **(C)** Dot-plot depicting the relative fold changes of CLEAR network gene transcripts in VPS35 KO cells relative to wild-type and VPS35-GFP rescue cells. Heatmap scale and FDR scores are indicated by colour of dots and rings, respectively. **(D)** Network analysis of significantly enriched cellular component gene sets. Red circles denote enriched categories, and blue circles denote depleted categories in VPS35 KO H4 cells. Circle size represents the number of enriched/depleted genes belonging to each gene set within the dataset. **(E)** Network analysis of significantly enriched KEGG pathway gene sets, presented as in (D). Circles are annotated with the number of enriched genes within each gene set. **(F)** Representative enrichment score plot genes enriched in the 'Parkinson's Disease' KEGG pathway, calculated by the GSEA software. **(G)** Table of selected significantly enriched KEGG pathway gene sets, depicting their rank, net enrichment score, size, and statistics. The full list of gene set enrichment is depicted in **Supplementary Data 8**.

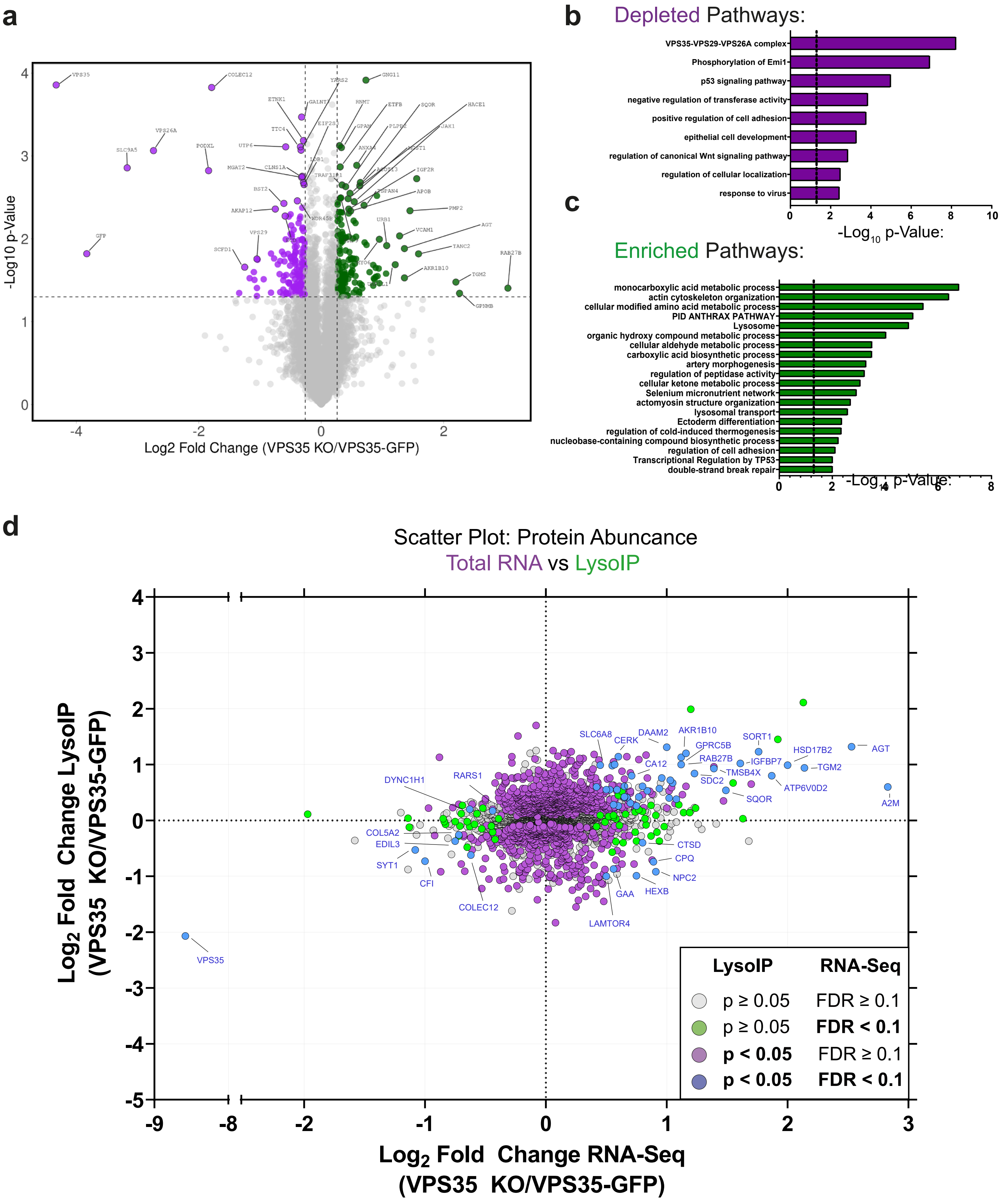
a
-Log10 p-Value
Log2 Fold Change (VPS35 KO/VPS35-GFP)
VPS35
COLEC12
VPS26A
SLC9A5
PODXL
GFP
GNG11
HACE1
RAB27B
GPNMB
TGM2
AGT
AKR1B10
b
Depleted Pathways:
VPS35-VPS29-VPS26A complex
Phosphorylation of Emi1
p53 signaling pathway
negative regulation of transferase activity
positive regulation of cell adhesion
epithelial cell development
regulation of canonical Wnt signaling pathway
regulation of cellular localization
response to virus
-Log10 p-Value:
c
Enriched Pathways:
monocarboxylic acid metabolic process
actin cytoskeleton organization
cellular modified amino acid metabolic process
PID ANTHRAX PATHWAY
Lysosome
organic hydroxy compound metabolic process
cellular aldehyde metabolic process
carboxylic acid biosynthetic process
artery morphogenesis
regulation of peptidase activity
cellular ketone metabolic process
Selenium micronutrient network
actomyosin structure organization
lysosomal transport
Ectoderm differentiation
regulation of cold-induced thermogenesis
nucleobase-containing compound biosynthetic process
regulation of cell adhesion
Transcriptional Regulation by TP53
double-strand break repair
-Log10 p-Value:
d
Scatter Plot: Protein Abuncance
Total RNA vs LysoIP
Log2 Fold Change LysoIP (VPS35 KO/VPS35-GFP)
Log2 Fold Change RNA-Seq (VPS35 KO/VPS35-GFP)
SLC6A8
DAAM2
AKR1B10
CERK
GPRC5B
SORT1
HSD17B2
AGT
CA12
RAB27B
IGFBP7
TGM2
TMSB4X
SDC2
ATP6V0D2
SQOR
A2M
DYNC1H1
RARS1
COL5A2
EDIL3
SYT1
CFI
COLEC12
CTSD
CPQ
NPC2
HEXB
GAA
LAMTOR4
VPS35
LysoIP
RNA-Seq
p ≥ 0.05 FDR ≥ 0.1
p ≥ 0.05 FDR < 0.1
p < 0.05 FDR ≥ 0.1
p < 0.05 FDR < 0.1

Supplementary Figure 7. Supplementary Whole Cell Proteome and Meta-Analysis

(A) Volcano plot of VPS35 KO/VPS35-GFP protein abundances in the whole cell proteome. 71 and 59 proteins were significantly enriched and depleted, respectively (Log2 fold change ± 0.26, $p < 0.05$), two-tailed paired t-tests. Magenta – proteins significantly depleted in VPS35 KOs, green – proteins significantly enriched in VPS35 KOs. **(B-C)** Pathway analysis of significantly depleted (B) or enriched (C) pathways in the VPS35 KO whole cell proteome, hypergeometric test. **(D)** Correlative analysis between RNA-Seq and LysoIP protein abundances. Scatter plot of VPS35 KO/VPS35-GFP RNA-Seq transcript abundances (x-axis) versus the protein abundances in the LysoIP (y-axis). Datapoints are coloured based on p-value scores in each experiment (green –significantly altered in RNA-Seq, magenta – significantly altered in LysoIP, blue –significantly altered in RNA-Seq and LysoIP), derived from two-tailed paired t-tests.

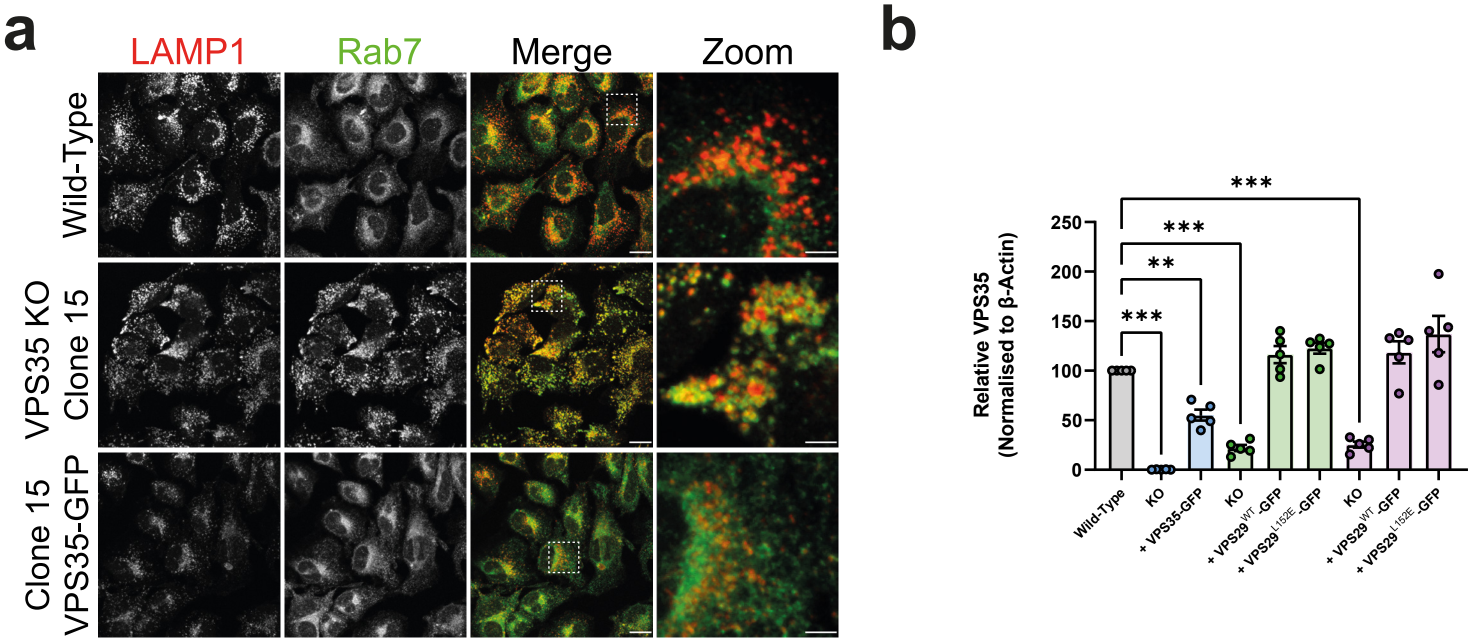
a
LAMP1
Rab7
Merge
Zoom
Wild-Type
VPS35 KO Clone 15
Clone 15 VPS35-GFP
b
Relative VPS35 (Normalised to β-Actin)
250
200
150
100
50
0
***
***
**
***
Wild-Type
KO
+ VPS35-GFP
KO
+ VPS29 WT -GFP
+ VPS29 L152E -GFP
KO
+ VPS29 WT -GFP
+ VPS29 L152E -GFP

Supplementary Figure 8. VPS35 KO H4 cells display hyper-recruitment of Rab7 to LAMP1-positive lysosomes

(A) VPS35 KO cells exhibit Rab7 hyper-recruitment to LAMP1-positive compartments. Immunofluorescence staining of wild-type, VPS35 KO Clone 15, and Clone15 VPS35-GFP-expressing cell stained with anti-Rab7 and -LAMP1 antibodies. Scale bar = 20 μm, zoom scale = 5 μm. **(B)** Quantification of VPS35 levels of indicated proteins were quantified relative to β-actin over n=5 independent experiments, displayed in Figure 2F. Means +/- SEM, one-sample t-tests with Holm-Šídák correction, adjusted p = 0.0008, 0.006, 0.0008, 0.3036, 0.0564, 0.0008, 0.3036, 0.3036.

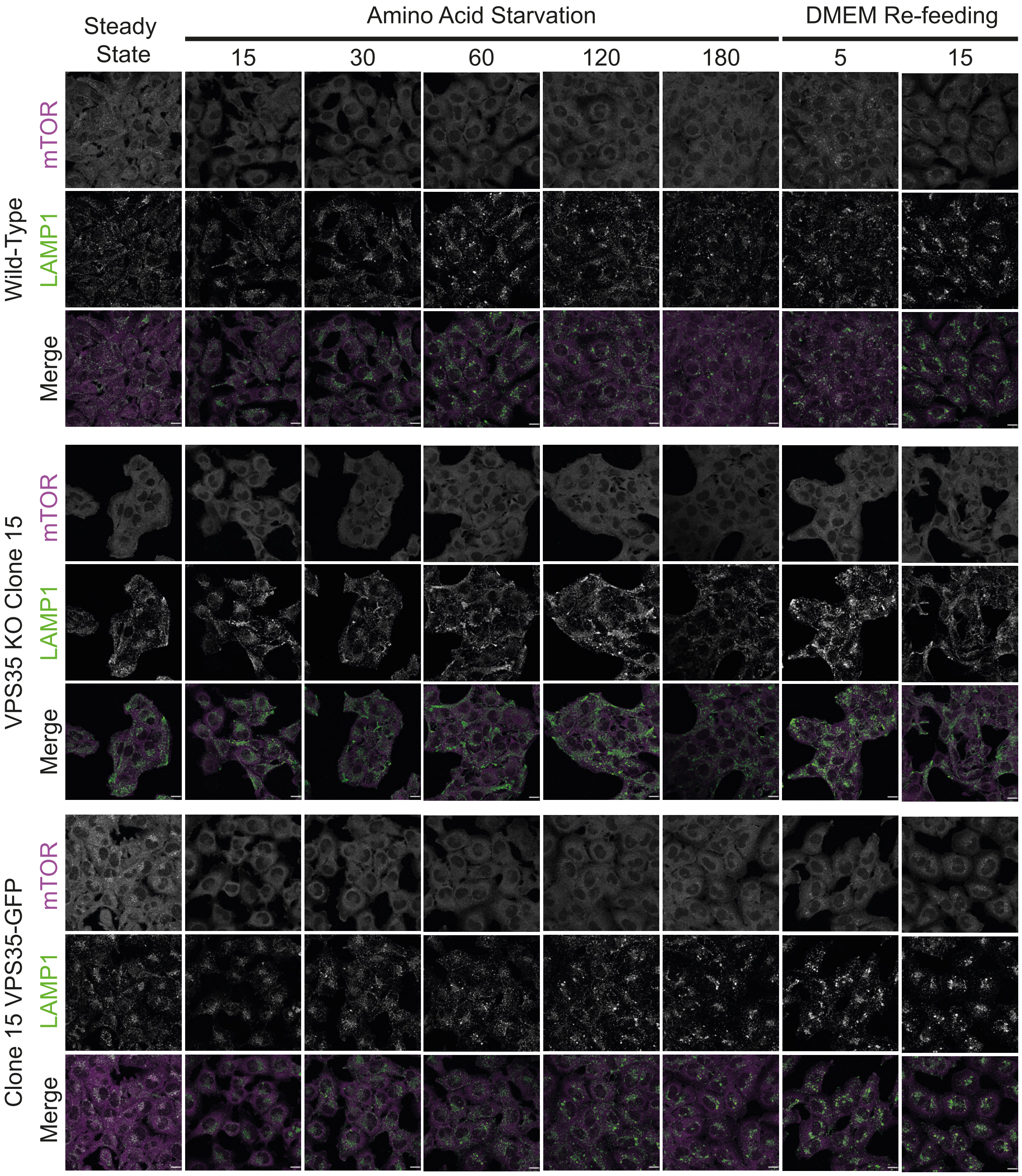
Steady State
Amino Acid Starvation
DMEM Re-feeding
15
30
60
120
180
5
15
Wild-Type
VPS35 KO Clone 15
Clone 15 VPS35-GFP
mTOR
LAMP1
Merge

Supplementary Figure 9. Impaired mTOR Recruitment in VPS35 KO H4 Cells

Lysosomal association of mTOR is unresponsive to amino acid starvation and re-feeding in VPS35 KO. Cells were amino acid starved prior to re-feeding in DMEM for the indicated time periods, then fixed and immuno-stained for mTOR and LAMP1. Scale bars = 20 μm. Data representative of 3 independent repeats.

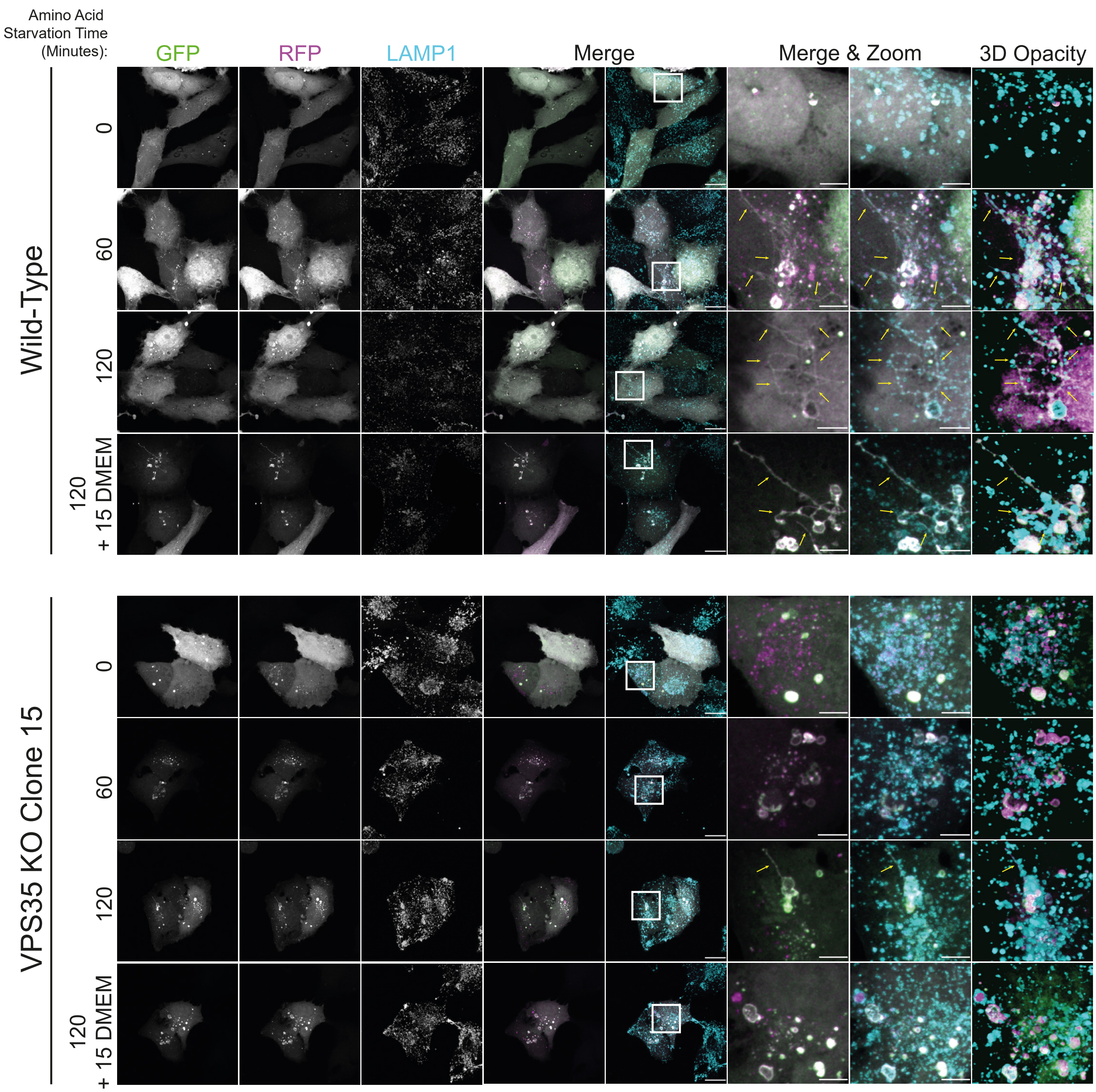

Amino Acid
Starvation Time
(Minutes):
GFP
RFP
LAMP1
Merge
Merge & Zoom
3D Opacity
Wild-Type
0
60
120
120
+ 15 DMEM
VPS35 KO Clone 15
0
60
120
120
+ 15 DMEM

Supplementary Figure 10. Defective Tubulation of Autophagic Lysosomes in VPS35 KO Cells

LC3-positive autophagic lysosomes display abundant tubulation in following starvation or re-feeding in wild-type cells, whereas these events are less common in VPS35 KO H4 cells. Cells were transfected with a GFP-RFP-LC3 dual reporter construct and starved prior to re-feeding in DMEM for the indicated time periods, then fixed and immuno-stained for LAMP1. Scale bars = 20 μm, zoom scale = 5 μm. Data representative of 3 independent repeats.
