# Supplementary material for: Multi-omic approach characterises the neuroprotective role of retromer in regulating lysosomal health
Source: Nat Commun. 2023 May 29;14:3086. doi: 10.1038/s41467-023-38719-8 (PMC10227043; doi:10.1038/s41467-023-38719-8)
Supplement: Supplementary file 2 — Description of Additional Supplementary Files [file 41467_2023_38719_MOESM2_ESM.pdf]

Description of Additional Supplementary Files

Supplementary Data Legends

Supplementary Data 1: Integrated Raw Proteomics and RNA-Seq Data from WildType, VPS35 KO and VPS35-GFP-expressing H4 Neuroglioma Cells. Data were not normalised. Output data is displayed for each protein across all independent experiments, including gene ontology classifications, peptide counts, protein coverage, Log2 fold change, twotailed unpaired t-test p-values and false discovery rate (FDR). This dataset was used for analysis of raw abundances in LysoIP and growth media 'secretome' datasets.

Supplementary Data 2: Integrated Normalised Proteomics and RNA-Seq Data from WildType, VPS35 KO and VPS35-GFP-expressing H4 Neuroglioma Cells. Data were normalised based on total peptide amount for each experiment. Output data is displayed for each protein across all independent experiments, including gene ontology classifications, peptide counts, protein coverage, Log2 fold change, twotailed unpaired t-test p-values and false discovery rate FDR. This dataset was used for analysis of relative abundances in LysoIP, surface biotinylation, total cell proteome and RNA-Seq datasets.

Supplementary Data 3: Pathway Enrichment Outputs from Proteomics Data. The top 20 enriched pathways for significantly enriched or depleted proteins across the datasets are provided, alongside p-value (hypergeometric test) and q-value (Benjamini-Hochberg correction) scores and the list of proteins within each category.

Supplementary Data 4: DisGeneNET Enrichment Outputs from Proteomics Data. Outputs from 3 comparisons are displayed: proteins significantly enriched in VPS35 KO LysoIP proteomics dataset; **significantly enriched proteins across all datasets; and significantly depleted proteins across all** datasets. In all cases, the raw output with all diseases is displayed, and the selected outputs displayed on graphs in the manuscript are shown in a separate tab. p-value (hypergeometric test) and q-value (BenjaminiHochberg correction) scores are provided.

Supplementary Data 5: Cellular Component Gene Ontology Analysis of the VPS35 KO 'Secretome'. Significantly enriched proteins from VPS35 KO samples (Log2 fold change > 1, $p < 0.05$), were analysed using PANTHER gene ontology software v16.0. All cellular component gene ontology categories are shown, with their respective fold enrichments and p-value scores, Fisher's exact test.

Supplementary Data 6: RNA-Seq Quantification of Global Transcript Abundances. RNA transcripts are displayed along with their abundances in wild-type, VPS35 KO or VPS35-GFP-expressing H4 cells. Log2 fold changes and FDR values are displayed.

Supplementary Data 7: RNA-Seq Quantification of CLEAR Network Genes. CLEAR network genes are displayed alongside their corresponding RNA-Seq Log2 fold changes and FDR values.

Supplementary Data 8: Gene Set Enrichment Analysis (GSEA) of RNA-Seq Data. Cellular component and Kyoto Encyclopaedia of Genes and Genomes (KEGG) pathway gene sets significantly enriched in VPS35 KO RNA-Seq datasets are displayed with enrichment scores and statistics, calculated by the GSEA software.

Supplementary Movie Legends

Supplementary Movie 1: Spinning disk live fluorescence microscopy of a wild-type H4 cell co-transfected with LAMP1-GFP and mCherry-LC3 showing autolysosome tubulation (merged GFP and mCherry channels)

Supplementary Movie 2: Spinning disk live fluorescence microscopy of a wild-type H4 cell co-transfected with LAMP1-GFP and mCherry-LC3 showing autolysosome tubulation (GFP only)

Supplementary Movie 3: Spinning disk live fluorescence microscopy of a wild-type H4 cell co-transfected with LAMP1-GFP and mCherry-LC3 showing autolysosome tubulation (mCherry only)

Supplementary Movie 4: Close up view of spinning disk live fluorescence microscopy of a wild-type H4 cell co-transfected with LAMP1-GFP and mCherry-LC3 showing autolysosome tubulation (merged GFP and mCherry channels)

Supplementary Movie 5: Close up view of spinning disk live fluorescence microscopy of a wild-type H4 cell co-transfected with LAMP1-GFP and mCherry-LC3 showing autolysosome tubulation (GFP only)

Supplementary Movie 6: Close up view of spinning disk live fluorescence microscopy of a wild-type H4 cell co-transfected with LAMP1-GFP and mCherry-LC3 showing autolysosome tubulation (mCherry only)

Supplementary Movie 7: Spinning disk live fluorescence microscopy of a VPS35 KO H4 cell co-transfected with LAMP1- GFP and mCherry-LC3 showing perturbed autolysosome dynamics (merged GFP and mCherry channels)

Supplementary Movie 8: Spinning disk live fluorescence microscopy of a VPS35 KO H4 cell co-transfected with LAMP1- GFP and mCherry-LC3 showing perturbed autolysosome dynamics (GFP only)

Supplementary Movie 9: Spinning disk live fluorescence microscopy of a VPS35 KO H4 cell co-transfected with LAMP1- GFP and mCherry-LC3 showing perturbed autolysosome dynamics (mCherry only)

Supplementary Movie 10: Close up view of spinning disk live fluorescence microscopy of a VPS35 KO H4 cell co-transfected with LAMP1- GFP and mCherry-LC3 showing perturbed autolysosome dynamics (merged GFP and mCherry channels)

Supplementary Movie 11: Close up view of spinning disk live fluorescence microscopy of a VPS35 KO H4 cell co-transfected with LAMP1- GFP and mCherry-LC3 showing perturbed autolysosome dynamics (GFP only)

Supplementary Movie 12: Close up view of spinning disk live fluorescence microscopy of a VPS35 KO H4 cell co-transfected with LAMP1- GFP and mCherry-LC3 showing perturbed autolysosome dynamics (mCherry only)
