# Supplementary material for: Multi-omic approach characterises the neuroprotective role of retromer in regulating lysosomal health
Source: Nat Commun. 2023 May 29;14:3086. doi: 10.1038/s41467-023-38719-8 (PMC10227043; doi:10.1038/s41467-023-38719-8)
Supplement: Supplementary file 23 — Reporting Summary [file 41467_2023_38719_MOESM23_ESM.pdf]

nature portfolio

Corresponding author(s): James L. Daly and Peter J. Cullen

Last updated by author(s): Apr 11, 2023

Reporting Summary

Nature Portfolio wishes to improve the reproducibility of the work that we publish. This form provides structure for consistency and transparency in reporting. For further information on Nature Portfolio policies, see our Editorial Policies and the Editorial Policy Checklist.

Statistics

For all statistical analyses, confirm that the following items are present in the figure legend, table legend, main text, or Methods section.

n/a	Confirmed	
☐	☒	The exact sample size (n) for each experimental group/condition, given as a discrete number and unit of measurement
☐	☒	A statement on whether measurements were taken from distinct samples or whether the same sample was measured repeatedly
☐	☒	The statistical test(s) used AND whether they are one- or two-sided<br>*Only common tests should be described solely by name; describe more complex techniques in the Methods section.*
☒	☐	A description of all covariates tested
☐	☒	A description of any assumptions or corrections, such as tests of normality and adjustment for multiple comparisons
☐	☒	A full description of the statistical parameters including central tendency (e.g. means) or other basic estimates (e.g. regression coefficient) AND variation (e.g. standard deviation) or associated estimates of uncertainty (e.g. confidence intervals)
☐	☒	For null hypothesis testing, the test statistic (e.g. F, t, r) with confidence intervals, effect sizes, degrees of freedom and P value noted<br>*Give P values as exact values whenever suitable.*
☒	☐	For Bayesian analysis, information on the choice of priors and Markov chain Monte Carlo settings
☒	☐	For hierarchical and complex designs, identification of the appropriate level for tests and full reporting of outcomes
☐	☒	Estimates of effect sizes (e.g. Cohen's d, Pearson's r), indicating how they were calculated

Our web collection on statistics for biologists contains articles on many of the points above.

Software and code

Policy information about availability of computer code

Data collection	Confocal microscopy: LAS AF, LAS X (Leica Microsystems). Western blot scanning: LI-COR Image Studio (LI-COR Biosciences). Mass Spectrometry: Proteome Discoverer v2.1 (Thermo Scientific), Xcalibur 2.1 (Thermo Scientific).
Data analysis	Immunofluorescence and electron microscopy image preparation and analysis: Volocity version 6.3.0 and ImageJ 1.5.3.<br>Proteomics and RNA-Seq analysis: Proteome Discoverer 2.1, r Studio, VolcanoseR (https://huygens.science.uva.nl/VolcaNoseR/), Metascape 3.5 (metascape.org), Cytoscape 3.9.1, Gene Set Enrichment Analysis software and MSigDB v7.2 (UCSanDiego, https://www.gsea-msigdb.org/gsea/index.jsp), PANTHER v16.0 (http://www.pantherdb.org/).<br>Western blot analysis: LI-COR Image Studio.<br>Statistical analysis and graph preparation: GraphPad Prism 9

For manuscripts utilizing custom algorithms or software that are central to the research but not yet described in published literature, software must be made available to editors and reviewers. We strongly encourage code deposition in a community repository (e.g. GitHub). See the Nature Portfolio guidelines for submitting code & software for further information.

Data

Policy information about availability of data

All manuscripts must include a data availability statement. This statement should provide the following information, where applicable:

- Accession codes, unique identifiers, or web links for publicly available datasets
- A description of any restrictions on data availability
- For clinical datasets or third party data, please ensure that the statement adheres to our policy

Source data are provided with this paper. The datasets generated during this study and minimum datasets required to interpret the data are included in the published article as Supplementary Data 1-8, and in public repositories. The mass spectrometry proteomics data generated in this study have been deposited in the ProteomeXchange Consortium via the PRIDE partner repository under accession code PXD041323 (https://proteomecentral.proteomexchange.org/cgi/GetDataset?ID=PXD041323). The RNA-Seq data generated in this study have been deposited in the NCBI Gene Expression Omnibus under accession code GSE223292 (https://www.ncbi.nlm.nih.gov/geo/query/acc.cgi?acc=GSE223292). Mass spectrometry data were searched against the human Uniprot database retrieved on 2021-01-14, and updated with additional annotation information on 2021-11-15. Unprocessed blots and statistical data are included as Source Data.

Human research participants

Policy information about studies involving human research participants and Sex and Gender in Research.

Reporting on sex and gender	*Use the terms sex (biological attribute) and gender (shaped by social and cultural circumstances) carefully in order to avoid confusing both terms. Indicate if findings apply to only one sex or gender; describe whether sex and gender were considered in study design whether sex and/or gender was determined based on self-reporting or assigned and methods used. Provide in the source data disaggregated sex and gender data where this information has been collected, and consent has been obtained for sharing of individual-level data; provide overall numbers in this Reporting Summary. Please state if this information has not been collected. Report sex- and gender-based analyses where performed, justify reasons for lack of sex- and gender-based analysis.*
Population characteristics	*Describe the covariate-relevant population characteristics of the human research participants (e.g. age, genotypic information, past and current diagnosis and treatment categories). If you filled out the behavioural & social sciences study design questions and have nothing to add here, write "See above."*
Recruitment	*Describe how participants were recruited. Outline any potential self-selection bias or other biases that may be present and how these are likely to impact results.*
Ethics oversight	*Identify the organization(s) that approved the study protocol.*

Note that full information on the approval of the study protocol must also be provided in the manuscript.

Field-specific reporting

Please select the one below that is the best fit for your research. If you are not sure, read the appropriate sections before making your selection.

☒ Life sciences ☐ Behavioural & social sciences ☐ Ecological, evolutionary & environmental sciences

For a reference copy of the document with all sections, see nature.com/documents/nr-reporting-summary-flat.pdf

Life sciences study design

All studies must disclose on these points even when the disclosure is negative.

Sample size	Sample size calculations were not performed. All experiments were performed using at least 3 independent biological repeats.
Data exclusions	No data were excluded from this study.
Replication	All quantified experiments were performed using at least 3 independent biological repeats. The reproducibility of findings is displayed by the individual datapoints on graphs, and all raw data is available in the source data.
Randomization	Samples were allocated into experimental groups based on their genotype (e.g. wild-type, VPS35 KO clones and VPS35-GFP clone rescues). Control conditions were used throughout the manuscript to validate the effects of VPS35 and VPS29 KO. Where possible, VPS35-GFP and VPS29-GFP rescues are used to this to demonstrate reversion of observed phenotypes by re-expression of the deleted gene.
Blinding	Blinding was not used in this study.

Reporting for specific materials, systems and methods

We require information from authors about some types of materials, experimental systems and methods used in many studies. Here, indicate whether each material, system or method listed is relevant to your study. If you are not sure if a list item applies to your research, read the appropriate section before selecting a response.

Materials & experimental systems

n/a	Involved in the study
☐	☒ Antibodies
☐	☒ Eukaryotic cell lines
☒	☐ Palaeontology and archaeology
☐	☒ Animals and other organisms
☒	☐ Clinical data
☒	☐ Dual use research of concern

Methods

n/a	Involved in the study
☒	☐ ChIP-seq
☒	☐ Flow cytometry
☒	☐ MRI-based neuroimaging

Antibodies

Antibodies used

Primary antibodies β-Actin (Sigma-Aldrich; A1978; clone AC-15; 1:2000 Western blot (WB)), APP (Abcam; Y188; ab32136; 1:200 immunofluorescence (IF)), ATP7A (Santa Cruz; D-9, sc-376467; 1:1000 WB), Cathepsin D (Proteintech; Clone, 21327-1-AP, 1:1000 WB), Cathepsin D (Merck; 219361; 1:200 IF), CI-MPR (Abcam; ab124767; clone EPR6599, 1:1000 WB, 1:400 immunofluorescence (IF)), EEA1 (Cell Signalling; 610456; clone 14; 1:200 IF), EGFR (Cell Signalling Technologies; 2232S; WB 1:1000), EGFR pY1068 (Cell Signalling Technologies; 3777S; WB1:1000), GFP (Roche; 11814460001; clones 7.1/13.1; 1:1000 WB), GLUT1 (Abcam; EPR3915; ab115730; 1:1000 WB; 1:50 IF), HA (Biolegend; 901502; Clone 16B12; 1:1000 WB, 1:200 IF), LAMP1 (Developmental Studies Hybridoma Bank; AB_2296838; clone H4A3; 1:400 IF) LAMP1 (Abcam; ab21470; 1:200 IF), LAMP1 (Cell Signalling Technologies; CS4H11; 1:1000 WB), p62 (SQSTM1) (BD Transduction Laboratories; 610832; 1:1000 WB, 1:200 IF) , Pmp70 (Sigma-Aldrich; 70-18; SAB4200181; 1:1000 WB), Rab7 (Abcam; EPR7589; ab137029; 1:200 IF), Rab27b (Proteintech; 13412-1-AP, 1:500 WB), TGN46 (Bio-Rad; AHP500G; 1:400 IF), TfnR (Santa Cruz; H68.4; sc-65883; 1:1000 WB), VPS29 (Santa Cruz; D-1; sc-398874; 1:500 WB), VPS35 (Abcam; ab157220; clone EPR11501(B); 1:1000 WB).

Secondary antibodies: For Western blotting, 680nm and 800nm donkey anti-mouse and anti-rabbit fluorescent secondary antibodies (Invitrogen, A-21057, A3275 - 1:20,000). For immunofluorescence, 405nm, 488nm, 568nm and 647nm AlexaFluor-labelled anti-mouse and anti-rabbit antibodies (Invitrogen, A32753, A32731, A10037, A10042, A32849, A32787, A32795 - 1:400). For mouse neuronal staining, donkey anti-rat 488 nm Alexa Fluor- or Cy3-conjugated secondary antibodies (Invitrogen or Jackson Immunoresearch, 712-156-150, 712-546-150) were used.

Validation

Validation was provided through the manufacturer's data sheet (see above for manufacturer and product code information) and/or the published use and validation of the relevant antibodies by our and other research groups.

Where possible, we technically validate antibodies for genes which we target for CRISPR-mediated knockouts. For example, we validate the specificity of our VPS35, VPS29 and GFP antibodies through immunoblotting of the respective knockout cell lysates relative to control and rescue conditions.

Eukaryotic cell lines

Policy information about cell lines and Sex and Gender in Research

Cell line source(s)	HeLa, HEK293T and H4 cells were originally sourced from ATCC.
Authentication	Authentication was provided by ATCC, and independently confirmed with Eurofins Genomics.
Mycoplasma contamination	Parental HeLa, HEK293T and H4 cells used to generate clonal KO cells were tested for mycoplasma contamination, with no contamination detected.
Commonly misidentified lines (See ICLAC register)	No commonly misidentified cell lines were used.

Animals and other research organisms

Policy information about studies involving animals; ARRIVE guidelines recommended for reporting animal research, and Sex and Gender in Research

Laboratory animals

All mice were used in the study were in a C57BL/6 genetic background. For both IUE and primary cortical neuron cultures, mothers were 8-12 weeks old. For primary cortical neuronal culture experiments, neurons were isolated at day E18.5. For IUE experiments, neocortical brain sections were taken at P14. The Vps35f/f mice were generated by injecting Vps35tm1a(EUCOMM)Hmgu embryonic stem (ES) cells (KO-first promoter-driven reporter-tagged insertion with conditional potential, purchased from International Mouse Phenotyping Consortium (IMPC), Germany) into the inner cell mass of C57BL/6J blastocysts. In Vps35tm1a(EUCOMM)Hmgu ES cell,

	the Vps35 gene contains loxP sites flanking exon 6. The injected blastocysts were then implanted into the uterus of pseudo-pregnant foster mothers for further mouse development.<br><br>The mice were maintained in cages with ad libitum access to water and standard food, which were located in a 12 h light/dark cycle animal room (with room temperature ~22°C, and humidity at 40-60%) at the animal facility of Case Western Reserve University (CWRU) with standard food and water provided and maintained on a 12 h dark/ light cycle.
Wild animals	Not applicable.
Reporting on sex	As it is a challenge task to identify sex in mouse embryos, the data were presented without sex-based analysis.
Field-collected samples	Not applicable.
Ethics oversight	The mouse breeding, maintenance, and experimental procedures were all approved by the Institute of Animal Care and Use Committees at the Case Western Reserve University, according to the National Institute of Health (NIH) guidelines.

Note that full information on the approval of the study protocol must also be provided in the manuscript.
